# ISG15 deficiency features a complex cellular phenotype that responds to treatment with itaconate and derivatives

**DOI:** 10.1002/ctm2.931

**Published:** 2022-07-17

**Authors:** Syed Fakhar‐ul‐Hassnain Waqas, Aaqib Sohail, Ariane Hai Ha Nguyen, Abdulai Usman, Tobias Ludwig, Andre Wegner, Muhammad Nasir Hayat Malik, Sven Schuchardt, Robert Geffers, Moritz Winterhoff, Sylvia Merkert, Ulrich Martin, Ruth Olmer, Nico Lachmann, Frank Pessler

**Affiliations:** ^1^ Research Group Biomarkers for Infectious Diseases, TWINCORE Centre for Experimental and Clinical Infection Research Hannover Germany; ^2^ Institute of Experimental Hematology Hannover Medical School Hannover Germany; ^3^ Department of Pediatric Pneumology, Allergology and Neonatology Hannover Medical School Hannover Germany; ^4^ Leibniz Research Laboratories for Biotechnology and Artificial Organs (LEBAO), Department of Cardiothoracic, Transplantation and Vascular Surgery (HTTG), REBIRTH‐Research Center for Translational and Regenerative Medicine Hannover Medical School Hannover Germany; ^5^ Biomedical Research in Endstage and Obstructive Lung Disease Hannover (BREATH) German Center for Lung Research (DZL), Hannover, Germany; ^6^ Department of Bioinformatics and Biochemistry, Braunschweig Integrated Centre of Systems Biology (BRICS) Technische Universität Braunschweig Braunschweig Germany; ^7^ Department of Bio and Environmental Analytics Fraunhofer Institute for Toxicology and Experimental Medicine Hannover Germany; ^8^ Genome Analytics Helmholtz‐Centre for Infection Research Braunschweig Germany; ^9^ Centre for Individualised Infection Medicine Hannover Germany; ^10^ Cluster of Excellence RESIST (EXC 2155) Hannover Medical School Hannover Germany; ^11^ Research Group Biomarkers for Infectious Diseases Helmholtz Centre for Infection Research Braunschweig Germany; ^12^ Current affiliation: Department of Medicine, Division of Allergy and Clinical Immunology Brigham and Women's Hospital, Harvard Medical School Boston Massachusetts USA

**Keywords:** 4‐octyl itaconic acid, apoptosis, ATP, branched chain amino acid amino transferase 1, dimethyl itaconic acid, induced pluripotent stem cells, inflammation, ISG15, itaconate, itaconic acid, mitochondrial biogenesis, NRF2, oxidative stress, pyroptosis, reactive oxygen species, ruxolitinib

## Abstract

**Background:**

Congenital ISG15 deficiency is a rare autoinflammatory disorder that is driven by chronically elevated systemic interferon levels and predominantly affects central nervous system and skin.

**Methods and results:**

We have developed induced pluripotent stem cell‐derived macrophages and endothelial cells as a model to study the cellular phenotype of ISG15 deficiency and identify novel treatments. *ISG15^–/–^* macrophages exhibited the expected hyperinflammatory responses, but normal phagocytic function. In addition, they displayed a multifaceted pathological phenotype featuring increased apoptosis/pyroptosis, oxidative stress, glycolysis, and acylcarnitine levels, but decreased glutamine uptake, BCAT1 expression, branched chain amino acid catabolism, oxidative phosphorylation, β‐oxidation, and NAD(P)H‐dependent oxidoreductase activity. Furthermore, expression of genes involved in mitochondrial biogenesis and respiratory chain complexes II–V was diminished in *ISG15^–/–^* cells. Defective mitochondrial respiration was restored by transduction with wild‐type ISG15, but only partially by a conjugation‐deficient variant, suggesting that some ISG15 functions in mitochondrial respiration require ISGylation to cellular targets. Treatment with itaconate, dimethyl‐itaconate, 4‐octyl‐itaconate, and the JAK1/2 inhibitor ruxolitinib ameliorated increased inflammation, propensity for cell death, and oxidative stress. Furthermore, the treatments greatly improved mitochondria‐related gene expression, BCAT1 levels, redox balance, and intracellular and extracellular ATP levels. However, efficacy differed among the compounds according to read‐out and cell type, suggesting that their effects on cellular targets are not identical. Indeed, only itaconates increased expression of anti‐oxidant genes *NFE2L2, HMOX1*, and *GPX7*, and dimethyl‐itaconate improved redox balance the most. Even though itaconate treatments normalized the elevated expression of interferon‐stimulated genes, *ISG15^–/–^* macrophages maintained their reduced susceptibility to influenza virus infection.

**Conclusions:**

These findings expand the cellular phenotype of human ISG15 deficiency and reveal the importance of ISG15 for regulating oxidative stress, branched chain amino acid metabolism, and mitochondrial function in humans. The results validate ruxolitinib as treatment for ISG15 deficiency and suggest itaconate‐based medications as additional therapeutics for this rare disorder.

## Graphical abstract

1. ISG15 deficiency triggers hyperinflammation, apoptosis/pyroptosis, and oxidative stress, while reducing mitochondrial respiration and biogenesis, branched chain amino acid catabolism, and viral infectivity.
2. Itaconates and the JAK1/2 inhibitor ruxolitinib ameliorate this dysregulated phenotype, identifying them as potential therapeutic options for this rare disorder.

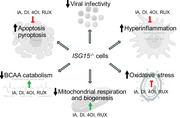

## 1 INTRODUCTION

Type I interferon (IFN‐I) signalling is activated by viral infection and results in tightly regulated antiviral innate immune responses.^1^ Inborn disbalance of this system can lead to type I interferonopathy, which is characterized by persistent upregulation of interferon‐stimulated genes (ISGs) resulting in autoinflammation and autoimmunity.^1^, ^2^, ^3^ Thus, monogenic type I interferonopathies can result from enhanced detection of intracellular nucleic acids leading to chronic induction of IFN‐I responses (familial chilblain lupus, Aicardi–Gutieres syndrome^4^), increased sensitization of IFN‐I signalling (STING associated vasculopathy with onset in infancy [SAVI]^5^), enhanced transcription of ISGs due to gain of function of STAT2 activation,^6^, ^7^ or due to loss of negative regulation of interferon α/β receptor (IFNAR) signalling by USP18. The latter may be caused by loss‐of‐function mutations in the *USP18* gene itself^8^ or in the gene encoding ISG15, which is required for stabilization of the USP18 protein in humans.^9^

ISG15 deficiency originally came to clinical attention because affected individuals had an unusual susceptibility to mycobacterial infections, which was subsequently found to be due to inadequate IFN‐γ synthesis resulting from lack of free (unconjugated) ISG15.^10^ Subsequently it was found that affected individuals may also, or predominantly, suffer from organ pathology resulting from chronic IFN‐I driven inflammation in CNS (seizures, intracranial calcifications^9^) and/or skin ulcerations^11^, ^12^. It remains to be clarified why one or the other clinical feature predominates in a given patient. Martin‐Fernandez et al. recently identified CD14^–^CD16^+^ monocytes as the peripheral blood cell type that drives chronic hyperinflammation and also showed that histiocytes in affected skin tissue are chronically activated.^11^ In the same report, we showed activation of IFN‐I signalling and hyperinflammation in IFN‐α stimulated vascular endothelial cells (ECs) and HaCaT keratinocytes.

ISG15 exists either as free molecule, both intracellularly and extracellularly, or in conjugates with lysine residues of target proteins reminiscent of ubiquitination (“ISGylation”). ISGylation is executed by ISG15‐activating enzymes, ISG15‐conjugating enzymes, and ISG15 E3 ligases.^13^ ISG15 expression and conjugation to targets is induced by a variety of processes, including infection, IFN‐α and ‐β signalling, ischemia, DNA damage, and aging. The current concept of human ISG15 deficiency is that IFN‐driven hyperinflammation and a reduced susceptibility to certain viral infections are the cardinal features of its cellular phenotype,^9^, ^14^ whereby the clinically intriguing lower viral infectivity may be explained by chronically elevated IFN‐I signalling and ISG expression. However, work in a variety of models has suggested that cellular functions of conjugated and free ISG15 go far beyond limiting IFN responses.^15^, ^16^ For instance, ISG15 has been implicated in regulating apoptosis,^17^ cell cycle,^18^ and autophagy,^13^ and cells from *ISG15^–/–^* mice show depressed mitochondrial respiration and reduced levels of mitochondrial reactive oxygen species (ROS).^19^ However, many of these studies were conducted in small animal models, which may not entirely reflect the human situation. This is exemplified by work using *ISG15^–/–^* mice, which do not manifest hyperinflammation or spontaneous skin lesions, likely because ISG15 is not required for USP18 stabilization in rodents.^15^ In addition, it is not known to what extent the various functions of ISG15 that have been described in a broad array of models actually contribute to the phenotype of a representative single model. This uncertainty would also suggest that treatments could be designed in a more targeted manner once the full phenotype is known.

Janus kinase (JAK) inhibitors such as ruxolitinib (RUX) are emerging as disease‐modifying treatment for type I interferonopathies, particularly for patients with severe disease.^20^ However, these medications are associated with high cost and may not be accessible to some patients or not be available in resource‐limited settings. In addition, they have not been evaluated for the treatment of ISG15 deficiency and it is not clear whether they would cover the whole spectrum of disease manifestations, since not all consequences of ISG15 loss may be due to lack of inhibition of IFNAR signalling but, rather, to absent ISGylation of protein targets other than USP18. Itaconic acid (IA) is a readily available, low‐cost dicarboxylic acid that has been used for decades in the synthesis of industrial polymers. It was recently identified as an immunomodulatory substance that occurs naturally in myeloid cells, notably activated macrophages.^21^, ^22^ Exogenously applied IA has well‐documented IFN‐reducing, immunomodulatory,^23^, ^24^, ^25^ and ROS‐reducing properties, which have been demonstrated, for example, in animal models of ischemic reperfusion injury (e.g.,^26^) and influenza A virus infection of human macrophages.^25^ Alkylated variants of IA such as dimethyl‐ and 4‐octyl‐IA (DI, 4OI) have more potent anti‐inflammatory and cytoprotective properties, likely due to greater electrophilicity.^24^ Consequently, there are ongoing efforts to develop optimized IA derivatives into disease‐modifying treatments for acute and chronic inflammatory diseases.

Considering the uncertainty of the full extent of the cellular phenotype of ISG15 deficiency and the need for additional treatment options, we have established a cellular disease model featuring cell types relevant to systemic inflammation (induced pluripotent stem cell [iPSC]‐derived macrophages and vascular endothelial cells) and cutaneous pathology (immortalized dermal fibroblasts and HaCaT keratinocytes). While placing the main focus on macrophages, we have used these diverse cell types to identify key phenotypic features resulting from loss of ISG15. Moreover, considering their well‐documented ability to reduce IFN‐I expression,^24^ we used this model to test whether itaconates and RUX could ameliorate aspects of the pathological phenotype.

## 2 MATERIALS AND METHODS

### 2.1 Cell culture

Wild‐type (WT) (MHHi001‐A)^27^ and *ISG15^–/–^* (MHHi001‐A‐3) hiPSCs^28^ were maintained on mouse embryonic fibroblasts (CF1 MEF), which were used as feeder cells as previously described^29^ or under feeder‐free conditions on Geltrex‐coated tissue culture flasks (TPP, Trasadingen, Switzerland) in E8 medium and passaged as single cells using Accutase™ (PAA, Pasching, Austria) with a seeding density of 3.6 × 10^4^ cells/cm^2^. For differentiation toward macrophages embryoid body (EB) formation was induced. hiPSCs were washed gently with phosphate‐buffered saline (PBS) and collagenase IV was then added to each well and incubated for 15–25 min in an incubator (37°C, 5% CO_2_) for detachment. Cells should be kept in fragments at this stage and resuspended in KO DMEM medium (without basic fibroblast growth facor). The cell suspension was transferred to a 6‐well suspension plate with 10 µM Rho‐associated kinase (ROCK) inhibitor (Y‐27632, Hölzel Diagnostica, Cologne, Germany) added. These plates were placed on a shaker at 85 rpm for 5 days. The medium was changed once or twice during this time and after 5 days, EBs > 200 µm diameter were picked using an upright low magnification microscope and transferred to a normal 6‐well plate containing X‐vivo differentiation medium I (Biozym (LONZA), Hessisch Oldendorf, Germany). 15–25 EBs were seeded per well and placed in an incubator for 8 days. After that, fresh X‐vivo differentiation medium I was added to the well, making sure that EBs were attached to the surface. At this stage EBs will start producing macrophages for up to 3–4 months, but the production rate will decrease with time. Differentiation toward ECs was performed as previously described.^11^, ^30^ Briefly, EC differentiation was induced by treatment with 25 ng/ml BMP4 (Bio‐Techne, Minneapolis, USA) and the WNT pathway activator CHIR 90221 (7.5 µM) in N2B27 medium (Thermo Fischer Scientific, Massachusetts, USA) for 2 days. From day 3 to 7, cultures were maintained in StemPro‐34 medium (Thermo Fischer Scientific, Massachusetts, USA) supplemented with 260 ng/ml rhVEGF‐A165 and 2 µM Forskolin (Sigma‐Aldrich, St. Louis, USA) with daily medium change. CD31+ cells were purified by MACS using CD31 MicroBead Kit (Miltenyi). hiPSC‐ECs were cultured on fibronectin (Corning, New York, USA) coated plates in ECGM‐2 medium (PromoCell, Darmstadt, Germany). hTert‐immortalized dermal fibroblasts from a healthy control and an ISG15‐deficient patient, as well as *ISG15^–/–^* fibroblasts transduced with WT or ΔGG *ISG15* were kindly provided by Dusan Bogunovic.^14^ In HaCaT cells the *ISG15* locus was ablated with the CRISPR/Cas9 system using two guide RNAs (gRNA) cloned separately into vector PX458_pSpCas9‐2A‐GFP (Addgene, Watertown, MA); gRNA1: GCGCAGATCACCCAGAAGAT; gRNA2: GGTAAGGCAGATGTCACAGG. 1 × 10^6^ cells were transfected with 1 µg of each targeting plasmid using Lipofectamine 3000 (Thermo Fischer Scientific, Massachusetts, USA) and green fluorescent protein (GFP) positive cells were isolated the next day by fluorescent activated cell sorting (FACS). The cell suspensions were then serially diluted (1:10) to obtain single cell clones, which were picked manually and expanded clonally. Homozygosity of the *ISG15* knock‐out was confirmed by PCR of genomic DNA, resulting in a single band of 378 bp in homozygous KO clones (fwd_TTTCTTCCGCTCACTCTGGG and rev_GTTCGTCGCATTTGTCCACC) and the absence of the 5′ (fwd_TTTCTTCCGCTCACTCTGGG, rev_GAGGATCTCAGGGGTGACCT) and 3′ (fwd_AGAGGACAGACAGGAGGGAG, rev_GTTCGTCGCATTTGTCCACC) junction of the WT allele, and by absence of ISG15 protein expression by western blot (Figure S3H).

### 2.2 RT‐qPCR analysis

mRNA was extracted using the Nucleospin RNA purification kit (Macherey‐Nagel, Düren, Germany) and reverse transcribed with PrimeScript™ kit (Takara, Shiga, Japan) according to the manufacturers’ protocols. Gene expression was analysed using the SensiFast™ SYBR® No‐ROX Kit (Bioline, Taunton, MA, USA) and a LightCycler480 instrument (Roche, Mannheim, Germany). The 2^–ΔΔCT^ method was used to measure relative mRNA expression of genes,^31^ using *HPRT* mRNA as internal control. Primer sequences are listed in Table S1.

### 2.3 Western blotting

Cells were lysed using RIPA buffer supplemented with freshly added protease and phosphatase inhibitors. Protein concentrations were measured by bicinchoninic acid (BCA) assay, equal amounts of protein were separated by SDS‐PAGE, transferred to a nitrocellulose membrane using semi‐dry blotting method, incubated with the respective antibodies, and bands visualized with Amersham enhanced chemiluminescence western blot detection reagent (GE Healthcare Science, Pittsburgh, USA). iNTAS imaging device (iNTAS Science Imaging, Göttingen, Germany) was used for imaging and measurement of relative band intensities. The following antibodies were used to detect the respective proteins: Human ISG15 (sc‐166755, 1:500); pSTAT1 (58D6, 1:1000); IFIT1 (14769S, 1:1000); MX1 (37849S, 1:1000); pAKT (4060S, 1:2000) (Cell Signaling Technology, MA, USA); β‐Actin (ab49900, 1:20 000) (Abcam, Cambridge, UK).

### 2.4 Enzyme linked immunosorbent assay

Cells were cultured in 12‐well plates, supernatants or cells were collected and sandwich ELISA were performed according to the manufacturers’ protocols using the following kits: IL‐1β (Biolegend; cat# 437004), IL‐10 (Biolegend; cat# 430601), IP‐10 (PeproTech GmbH; cat# 900‐K39), and GSK3β (Abnova/Fisher scientific; cat# KA2908) according to the manufacturers’ protocols.

### 2.5 Mitochondrial ROS measurement

Cells were seeded at a density of 2 × 10^5^ cells per well in a 12‐well plate, incubated for 5 min with medium containing 5 µM of MitoSOX ™ Red (mitochondrial superoxide indicator, Invitrogen, cat# M36008) and then washed with PBS. Cells were then resuspended in cold PBS and mitochondrial ROS was measured via the phycoerythrin (PE) channel using a BD™ LSR‐II flow cytometer.

### 2.6 Phagocytosis assay

Cells were cultured in a 96‐well plate at a density of 2 × 10^4^ cells per well. PBMCs from healthy volunteers,^25^ WT, and *ISG15* ^*–/–*^ macrophages were kept in RPMI media supplemented with M‐CSF for 5 days, for terminal differentiation into the M2 activation state. After removal of nonadherent cells, phagocytosis assay was performed according to the manufacturers’ protocols (Phagocytosis Assay–Zymosan Substrate kit; Abcam, ab211156).

### 2.7 ATP measurement assay

Cells were seeded in a 12‐well plate at a density of 2 × 10^5^ cells per well. After completion of the respective experiment, supernatants were collected for extracellular ATP measurement and RIPA buffer was added to obtain cell lysates for intracellular ATP measurements. ATP was measured according to the manufacturers’ protocol using ATP Determination Kit (ThermoFisher Scientific, A22066). Luminescence was measured using a luminometer (Centro XS^3^ LB960, Berthold Technologies, Bad Wildbad, Germany).

### 2.8 Single‐cell RNA sequencing

Macrophages were isolated from EB‐containing culture by FACS using CD11b, CD14, CD45 and CD163 as macrophage markers.^32^ Cells were washed with 1x PBS and 1x binding buffer and incubated for 15 min with propidium iodide (PI) solution (Apoptosis Detection Kit, eBioscience, cat# 88‐8005‐74). Live and single cells were sorted by FACS based PI staining and single cell suspensions prepared in 1x PBS (10x Genomics). The single cell suspension was loaded onto a Chromium Single Cell Controller (10x Genomics). Barcoding and cDNA synthesis were performed according to the manufacturer's instructions. Quality was checked using the Agilent Bioanalyzer High Sensitivity Assay. Libraries were sequenced on Illumina NovaSeq 6000 2 × 50 paired‐end kits using the following read lengths: 26 bp Read1 for cell barcode and Unique Molecular Identifier (UMI), 8 bp I7 index for sample index and 89 bp Read2 for transcript. Cell Ranger 1.3 (http://10xgenomics.com) was used to process Chromium single cell 3′ RNA‐seq output. First, “cellranger mkfastq” demultiplexed the sequencing samples based on the 8 bp sample index read to generate fastq files for the Read1 and Read2, followed by extraction of 16 bp cell barcode and 10 bp UMI. Second, “cellranger count” aligned the Read2 to the mouse reference genome (mm10) using STAR. Then, aligned reads were used to generate data matrix only when they have valid barcodes and UMI, map to exons (Ensembl GTFs GRCm38.p4) without PCR duplicates. Valid cell barcodes were defined based on UMI distribution. Cells were removed when they had <500 unique feature counts and >25% reads mapped to mitochondrial expression genome (Figure S17). After filtering and doublet removal, the following cell numbers remained: WT con = 1894 cells, WT IFN‐α = 741, WT IFN‐α + IA = 2590, WT IFN‐α + 4OI = 3045, KO con = 2364, KO IFN‐α = 2107, KO IFN‐α + IA = 2378 and KO IFN‐α + 4OI = 2279.

Single‐cell gene expression data analysis including filtering, normalization, and clustering was processed using Seurat V3.1 (https://satijalab.org/seurat/) in the R environment for computing (www.bioconductor.org). Raw counts were normalized by the global‐scaling normalization method “LogNormalize”.

### 2.9 Bulk RNA sequencing

Total RNA was isolated using Nucleospin RNA purification kit (Macherey‐Nagel, Düren, Germany). RNA quality was checked on an Agilent Technologies 2100 Bioanalyzer (Agilent Technologies; Waldbronn, Germany), and only samples with RNA Integrity Number ≥8 were used. The RNA sequencing library was generated from 100 ng total RNA using NEBNext® Single Cell/Low Input RNA Library according to the manufacturer´s protocols (New England BioLabs). The libraries were sequenced on an Illumina NovaSeq 6000, using the NovaSeq 6000 S1 Reagent Kit (100 cycles, paired‐end run 2 × 50 bp) with an average of 5 × 10^7^ reads per sample. Sequencing reads were mapped against *Homo sapiens* hg38 reference genome via STAR 2.7.3a tool.

### 2.10 Small non‐coding RNA sequencing

Using aliquots from the same total RNA used for long RNAseq, quality and integrity of RNA was checked on an Agilent Technologies 2100 Bioanalyzer (Agilent Technologies; Waldbronn, Germany). NEBNext® Multiplex Small RNA Library Prep Set for Illumina was used for library preparation. 200 ng of total RNA was used as input and the libraries were sequenced on an Illumina NovaSeq 6000 using the NovaSeq 6000 S1 Reagent Kit (100 cycles, paired end run 2 × 50 bp) with an average of 2 × 10^7^ reads per RNA sample. FASTAQ files were then obtained to annotate the transcripts as described in the OASIS 2.0 web tool tutorial.^33^ Briefly, FASTAQ files were compressed by OASIS compressor uploaded for annotation using the OASIS web tool and reference genome (*Homo sapiens* – hg38). Potential targets of the miRNAs were predicted using the TargetScan, miRDB and miRTarBase programs using the miRWalk 3.0 tool, keeping only those targets that were predicted by all three programs. Potential targets were uploaded to the WebGestalt platform for estimation of enriched pathways, using a threshold of false discovery rate (FDR) of <.05.

### 2.11 Seahorse Mito Stress assay

SeahorseXF Mito Stress assay (Seahorse XFe/XF analyser, Agilent) was used to measure mitochondrial function. It is a plate‐based live‐cell assay that measures oxygen consumption rate (OCR) in real time. Briefly, cells were seeded in a Seahorse XF cell culture microplate and subjected to IFN‐α stimulation and/or treatment as indicated. Cells were washed with Seahorse XF DMEM medium (supplemented with 8 mM glucose, 1 mM pyruvate and 2 mM glutamine, pH 7.4) and then kept in this medium in a non‐CO_2_ incubator at 37°C for 60 min. The Seahorse cartridge has four injection ports (A‐D). Injection solutions of 1 µM oligomycin (port A), 1.5 µM carbonyl cyanide‐4 (trifluoromethoxy) phenylhydrazone (FCCP) (port B), 1 µM rotenone (port C), and 1 µM antimycin (port D) were prepared in Seahorse medium and added to the respective ports once incubation of the cartridge was complete. The cartridge was then loaded into the Seahorse instrument and calibrated for 15–20 min. Following calibration, the cell culture microplate was loaded to run the assay. Once the assay was complete, WAVE software generated the report, which was then exported to an Excel file.

### 2.12 Crystal violet staining

Crystal violet staining was used to quantify the cells in each well after the completion of Seahorse assay. Briefly, cells were washed twice with PBS, 300 µl of crystal violet staining solution was added to each well, and the plate was incubated for 10 min. Afterward, it was washed three times with PBS, and 200 µl of 1% SDS was added to each well and the plate was kept on a rocking shaker for 30 min. Last, 100 µl of experimental lysate was transferred to a new 96‐well plate and absorbance at 570 nm was measured with a spectrophotometer. Seahorse data were normalized with OD of each well for final calculations of OCR.

### 2.13 Fluorometric assay

A caspase multiplex fluorometric assay kit (Abcam; cat# ab219915) was used to measure caspase activity to detect cells undergoing apoptosis. Briefly, cells were seeded in a 96‐well plate and after completion of the experiment, medium was removed and assay loading solution (caspase substrate and assay buffer) was added to each well. The plate was then incubated in the dark for 60 min and fluorescence was measured with a fluorescence microplate reader.

### 2.14 Glutathione measurements

GSH/GSSG Ratio Detection Assay Kit II (Abcam, ab205811) was used to detect reduced (GSH) and oxidized (GSSG) glutathione levels. Cells were seeded in a 12‐well plate and after completion of the experiment, medium was removed and cells were washed with cold PBS. Cells were lysed, homogenized for 15 min, and clear supernatant was collected and transferred to a new tube. For GSH detection, 50 µl of GSH assay mixture was added to standards and samples. For GSSG measurement, 50 µl of total glutathione assay mixture was added to standards and samples. After incubation of 15 min, fluorescence was measured at Ex/Em = 490/520 nm with fluorescence microplate reader.

### 2.15 FAM‐FLICA caspase‐1 assay

To detect pyroptosis, the FAM‐FLICA caspase‐1 assay kit (Biozol/Immunochemistry; cat# ICT‐98) was used according to the manufacturer's protocol. Briefly, cells were grown in 12‐well plates at a density of 2 × 10^5^ cells per well. Once IFN‐α stimulation and treatment were complete, cells were collected in FACS tubes and washed with 10x apoptosis wash buffer. FLICA was added to each sample at 1:30 dilution and samples were incubated for 1 h. Afterward, samples were washed and resuspended in wash buffer and analyzed using a BD™ LSR‐II flow cytometer (BD Biosciences, San Jose, CA).

### 2.16 LDH release assay

Cytotoxicity Detection Kit^PLUS^ (Roche, Mannheim, Germany) was used to assess LDH release as a measure of cytotoxicity. Cells were seeded in a 96‐well plate and after the completion of treatment, 5 µl of lysis solution was added and incubated for 15 min. Meanwhile catalyst and dye solution were mixed to make a reaction mixture, and 100 µl of this reaction mixture was added to each well containing supernatants and incubated for 30 min in the dark at room temperature. Last, 50 µl of stop solution was added to each well and after shaking for 10 s, OD was measured at 490 nm using an ELISA plate reader.

### 2.17 MTT assay

Yellow tetrazolium salt (3‐(4,5‐dimethylthiazol‐2‐yl)‐2,5‐diphenyltetrazolium bromide, or MTT) kit (Life Technologies, Carlsbad, California) was used to measure cell respiration as a measure of cell viability according to the manufacturer's protocol. Briefly, cells were seeded in a 96‐well plate and after completion of the treatment 100 µl of MTT labelling reagent (final concentration 0.5 mg/ml) was added to the cells and incubated at 37°C for 60 min. MTT was removed from the wells and 100 µl of solubilization solution (DMSO) was added to each well and, once purple formazan crystals were formed and completely solubilized, the plate was measured at 540 nm wavelength using a spectrophotometer.

### 2.18 Microarray analysis

RNA was extracted using the RNeasy kit (Qiagen), RNA quality was checked with a Bioanalyzer 2100 (Agilent Technologies) and samples with RNA integrity number >7 were used for microarray analysis. Total RNA/Poly‐A RNA Control Mixture was prepared by adding poly‐A RNA controls supplied by Affymetrix. This poly‐A RNA was then used to prepare double stranded cDNA. In the next step, complimentary RNA (cRNA) was synthesized and amplified by in vitro transcription of the ds‐cDNA template using T7 RNA polymerase. Enzymes, salts, inorganic phosphates, and unincorporated nucleotides were removed to prepare the cRNA for 2nd‐cycle ds‐cDNA synthesis. The quality and yield of cRNA was assessed. Later, sense‐strand cDNA was synthesized by reverse transcription of cRNA. The cRNA template was hydrolysed leaving ds‐cDNA, which was then purified. cDNA was fragmented and then labelled by terminal deoxynucleotidyl transferase (TdT) using the Affymetrix proprietary biotin‐linked DNA Labelling Reagent. Cartridge Array Hybridization was conducted on the GeneChip® Instrument (Affymetrix) using Thermo Fisher Scientific microarrays (Clariom™ S Assay, human) [252‐254]. Files were analyzed for differential gene expression using the Transcriptome Analysis Console (TAC4.0.2) Software (ThermoFisher Scientific). Metaboanalyst software (https://www.metaboanalyst.ca/) was used for differential expression analysis. TAC files were also processed for functional enrichment analysis using WEB‐based GEne SeT AnaLysis Toolkit (Webgestalt, http://www.webgestalt.org/), defining enriched pathways as those having a false discovery rate (FDR) <.05. KEGG gene sets were downloaded from GSEA online portal. R‐Bioconductor package “fgsea” was used to pre‐rank the differentially expressed genes and annotate them against different pathways.

### 2.19 Targeted metabolomics by mass spectrometry

The mass spectrometric measurements for the targeted metabolomics screen were performed on an AB SCIEX 5500 QTrap™ mass spectrometer (AB SCIEX, Darmstadt, Germany) using the MxP® Quant500 kit (Biocrates Life Sciences, Innsbruck, Austria), following the manufacturer's protocols (https://biocrates.com/mxp‐quant‐500‐kit). 5 × 10^6^ WT or *ISG15* ^*–/–*^ dermal fibroblasts were used per sample and processed according to Biocrates application note 2001‐1, using extraction buffer containing 10 mM phosphate buffer/85% ethanol and adding three freeze‐thaw‐sonication cycles to maximize cell lysis.^34^ Output files were generated by MetIDQ™ software (Biocrates Life Sciences, Innsbruck, Austria) and analyzed further using Metaboanalyst software (https://www.metaboanalyst.ca/).

### 2.20 ^13^C stable isotope assisted metabolic flux assay

Cells were seeded in 6‐well plates and 25 mM glucose (^12^C or ^13^C_6_) and 4 mM of glutamine (^12^C or ^13^C_5_) containing medium was added to each well. After IFN‐α stimulation, the cells were washed with 0.9% NaCl, and 400 µl of −20°C methanol (Chromasolve) was added to the cells. Subsequently, 400 µl of Millipore water containing 1 µg/ml D6 glutaric acid (internal standard) was added. Cells were scraped and the suspension was transferred to an Eppendorf tube containing 400 µl of −20°C chloroform (Chromasolve). The tubes were vortexed for 20 min at 4°C and then centrifuged for 5 min at 17 000 g. 250 µl of the polar (top) phase was transferred to a mass spec (MS) vial. Samples were dried using a refrigerated vacuum centrifuge set to −4°C. Before removing the vials, they were warmed to room temperature to avoid condensation. Samples were then analyzed by gas chromatography‐mass spectrometry (GC‐MS).

### 2.21 GC‐MS measurement

Metabolites were derivatized using an Axel Semrau Chronect Robotic Pal TRC. Dried polar metabolites were dissolved in 15 µl of 2% methoxyamine hydrochloride in pyridine at 55°C under shaking. After 90 min, an equal volume of N‐(tert‐butyldimethylsilyl)‐N‐methyltrifluoroacetamide (MTBSTFA, Sigma‐Aldrich) was added and held for 30 min at 55°C under continuous shaking. One microliter sample was injected into an SSL injector at 270°C in splitless mode. GC‐MS analysis was performed using an Agilent 7890A GC equipped with a 30‐m ZB‐35 + 5‐m Duraguard capillary column (Phenomenex). Helium was used as carrier gas at a flow rate of 1 ml/min. The GC oven temperature was held at 100°C for 2 min, increased to 300°C at 10°C/min, and finally held for 4 min at 300°C. The GC was connected to an Agilent 5975C inert XL MSD, operating under electron ionization at 70 eV. The MS source was held at 230°C and the quadrupole at 150°C. The detector was operated in selected ion monitoring. The total run time per sample was 26.00 min.

### 2.22 Mass isotopomer distribution analysis

All GC‐MS chromatograms were processed using MetaboliteDetector.^35^ Chemical formulas for mass isotopomer distribution (MID) determination were taken from Reference.^36^ Weighted carbon contribution was calculated with the following formula: 1/*n*∗ΣMi*i, where n is the number of carbons of the molecule of interest and M_i_ the *i*th mass isotopomer.

### 2.23 Sample preparation for IA measurements by HPLC‐MS/MS

iPSC‐derived macrophages were cultivated in 6‐well plates as described above and stimulated with IFN‐α for 24 h at 37°C and 5% CO_2_. Subsequently, the medium was aspirated and cells were washed once with 1xPBS. After carefully removing the supernatant, cells were extracted with 1000 µl of extraction reagent (methanol/acetonitrile/water; 2/2/1; v/v; spiked with 0.1 µM ^13^C_2_‐citrate and ^13^C_5_‐IA, 0.2 µM ^13^C_6_‐cis‐aconitate and 1 µM ^13^C_3_‐lactate as internal standards). The suspensions were transferred to 2 ml safe‐lock reaction tubes (0030120094 Eppendorf), vortexed for 30 s and frozen at −20°C overnight to complete protein precipitation. The subsequent sample preparation and HPLC‐MS/MS assay using a Kinetex C18 column (00D‐4462‐Y0, Phenomenex) on a Nexera chromatography system (Shimadzu, Japan) coupled to a QTRAP5500 triple quadrupole/linear ion trap mass spectrometer (Sciex, Framingham, MA, USA) was performed essentially as described in reference.^37^

### 2.24 Influenza A virus infection

The 2009 pandemic field isolate (A/Giessen/06/09 [H1N1]) was used for viral infection. Briefly, macrophages were seeded at a density of 2 × 10^5^ cells/well in 12‐well plates and incubated overnight at 37°C. The following day, cells were infected with influenza A virus at a multiplicity of infection (MOI) of 1. Cells were kept in DMEM media containing virus (without FCS) for 2 h and then washed with PBS to remove unbound virus and fresh medium containing all the treatments was added to the respective wells. Once the experiment was complete, RA1 lysis buffer was used to collect the cells.

### 2.25 Statistical analysis

All experiments featured 3–4 biological replicates. Data are expressed as means ± standard error of mean (SEM) unless stated otherwise. GraphPad Prism 8.0.2 was used for statistical analyses. One‐way ANOVA with Tukey's test to adjust for multiple testing was performed to assess significant difference of means among three or more groups. Student's *t*‐test was performed to assess significance of differences in means between two groups. P values <.05 were considered significant, using the following abbreviations: *, *p* < .05; **, *p* < .01; ***, *p* < .001; ****, *p* < .0001.

DESeq2 tool (https://bioconductor.org/packages/release/bioc/html/DESeq2.html) was used for differential expression analysis of bulk RNAseq data. Microarray data were analyzed using Transcriptome Analysis Console (TAC4.0.2) Software (ThermoFisher Scientific).

## 3 RESULTS

### 3.1 Type I IFN signature and hyperinflammation in ISG15 deficiency

**FIGURE 1:**
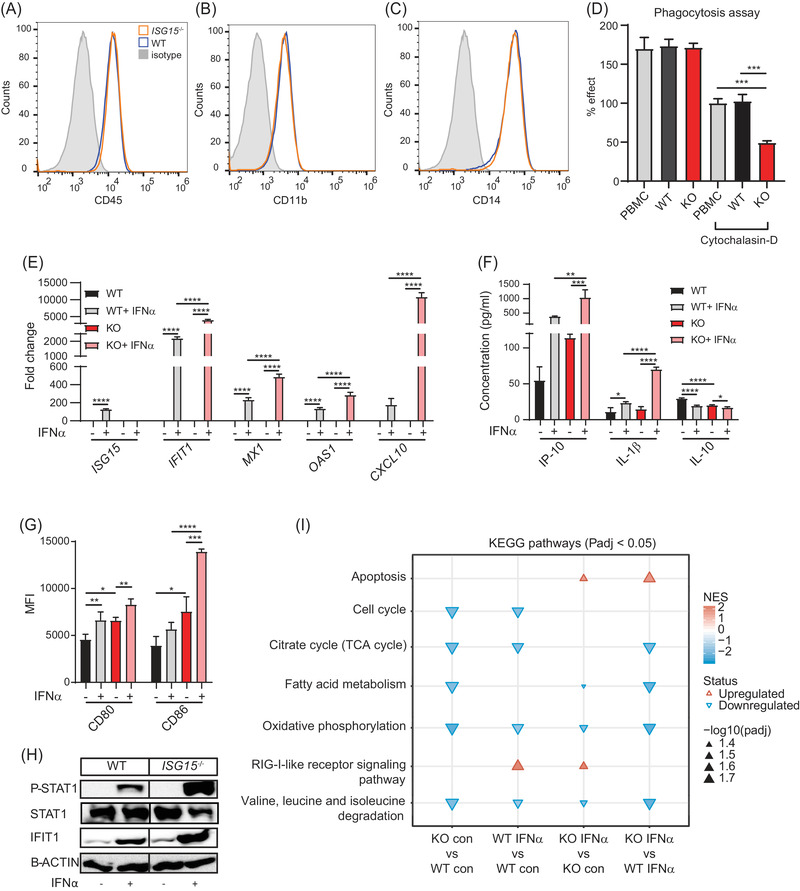
Phenotypic and functional characterization of iPSC‐derived macrophages. (A**–**C) Flow cytometry analysis demonstrating similar expression of CD45 (A) and the classic macrophage markers CD11b (B) and CD14 (C) on WT and *ISG15^–/–^* (KO) iPSC‐derived macrophages. (D) Phagocytosis assay demonstrating similar phagocytic activity of human PBMC and WT and *ISG15^–/–^* iPSC‐derived macrophages. The phagocytosis inhibitor cytochalasin‐D was added as positive control. 100% = cytochalasin‐D treated PBMC. (E‐H) Pro‐inflammatory phenotype of *ISG15^–/–^* iPSC‐derived macrophages. WT and *ISG15^–/–^* macrophages were stimulated with 1000 IU/ml of IFN‐α for 24 h as indicated on the *x*‐axes. (E) Increased expression of *ISG15, IFIT1, MX1, OAS1*, and *CXCL10* mRNA in *ISG15^–/–^* cells after IFN‐α stimulation (RT‐qPCR). (F) Higher concentrations of IP‐10 and IL‐1β, but lower concentrations of IL‐10 in cell culture supernatants of *ISG15^–/–^* cells (ELISA). (G) Higher cell surface expression of CD80 and CD86 on *ISG15^–/–^* cells with and without IFN‐α stimulation (flow cytometry; MFI, mean fluorescent intensity). (H) Higher levels of phosphorylated STAT1 (P‐STAT1) and IFIT1 in *ISG15^–/–^* cells after IFN‐α stimulation (western blot of cell lysates). (I) Gene set enrichment analysis based on KEGG pathways (FDR < .05) for the pairwise comparisons indicated below the figure. Selection of the most relevant pathways in the enrichment analysis is shown in Figure S5E. The normalized enrichment score (NES) is calculated by ranking gene expression differences and normalizing for size of each gene set. Significance of enrichment/depletion is indicated by the size of the triangles. Abbreviations: con, unstimulated; IFNα, IFNα stimulated. *, *p* < .05; **, *p* < .01; ***, *p* < .001; ****, *p* < .0001, one‐way ANOVA followed by Tukey's post hoc test

WT and *ISG15^–/–^* macrophages were validated phenotypically and functionally in that they expressed CD45, CD11b, and CD14. In addition, they demonstrated phagocytic activity similar to M2‐type macrophages derived from human PBMC, which could be inhibited by cytochalasin‐D, a classic inhibitor of phagocytosis (Figure 1A–D).

Increased IFN‐I expression is a cardinal sign of ISG15 deficiency.^9^ To verify the presence of an IFN‐I signature and associated hyperinflammation in iPSC‐derived macrophages, we stimulated *ISG15^–/–^* and WT cells with IFN‐*α*2b. Indeed, expression of ISG mRNAs *ISG15, IFIT1*, *MX1, OAS1*, and *CXCL10* was higher in *ISG15^–/–^* than WT macrophages (Figure 1E and Figure S1A). Also, in cell culture supernatants of *ISG15^–/–^* cells there were higher levels of the *CXCL10* gene product, IP10 (a pro‐inflammatory chemokine), and the classic pro‐inflammatory cytokine IL‐1β, whereas the anti‐inflammatory cytokine IL‐10 was down‐regulated (Figure 1F). Moreover, expression of the pro‐inflammatory surface markers CD80 and CD86 was higher on unstimulated and IFN‐α stimulated *ISG15^–/–^* cells, and intracellular levels of phosphorylated STAT1 (P‐STAT1 = activated STAT1) and IFIT1 proteins (Figure 1 G,H) were higher in stimulated *ISG15^–/–^* cells. Thus, the *ISG15^–/–^* iPSC‐derived macrophages possessed the strong IFN‐I signature which is characteristic of patients affected by ISG15 deficiency. The *ISG15^–/–^* macrophages also demonstrated a more general hyperinflammatory phenotype in that the induction of *IFIT1* and *CXCL10* mRNA and IL‐1β protein by LPS was significantly stronger than in WT cells, even though the absolute degree of induction of *IFIT1* and *CXCL10* mRNA was considerably less in both WT and *ISG15^–/–^* than after IFN‐α stimulation (Figure S1C–E).

Additionally, we assessed inflammation in iPSC‐derived ECs (Figure S2), the transformed keratinocyte cell line HaCaT (Figure S3), and immortalized dermal fibroblasts (Figure S4) and found the expected hyperinflammatory phenotype of *ISG15^–/–^* cells in all cases.

### 3.2 Transcriptomic profiling and functional enrichment analysis reveals additional phenotypic features of *ISG15^–/–^* cells

We then used transcriptome profiling to search for additional phenotypic differences between *ISG15^–/–^* and WT macrophages, both at the bulk and single‐cell level. Principle component analysis (PCA) of transcriptomes obtained by bulk RNAseq revealed clear differences between *ISG15^–/–^* and WT cells both with and without IFN‐α stimulation (Figure S5B). Expression of the enzyme that catalyses IA synthesis during macrophage activation (ACOD1) is IFN‐inducible.^38^ In agreement with this, the RNAseq analysis revealed that IFN‐α stimulation results in higher levels of *ACOD1* mRNA in *ISG15^–/–^* macrophages,  which was corroborated by higher intracellular IA concentration (Figure S5C,D).

Considering their key roles in regulating gene expression, we analyzed the miRNA data obtained by small RNA sequencing of the same samples. Indeed, a PCA showed distinct miRNA expression patterns in *ISG15^–/–^* and WT macrophages with and without IFN‐α stimulation (Figure S6A). PCA of the mRNA microarray data from iPSC‐derived ECs also showed distinct expression patterns in *ISG15^–/–^* and WT cells with and without IFN‐α stimulation (Figure S7A). Thus, loss of ISG15 led to distinct transcriptome patterns in both macrophages and ECs, and a distinct *ISG15^–/–^* transcriptomic phenotype was apparent even without IFN‐α stimulation.

We then used gene set enrichment analysis (GSEA) based on KEGG pathways to identify pathways whose dysregulation might contribute to the *ISG15^–/–^* phenotype. Compared to IFN‐α stimulated WT macrophages, IFN‐α stimulated *ISG15^–/–^* macrophages featured increased *apoptosis*, but depletion of *cell cycle*, *fatty acid metabolism*, *oxidative phosphorylation*, *PPAR signalling*, *purine metabolism*, *pyrimidine metabolism*, *pyruvate metabolism*, *TCA cycle*, and *valine, leucine and isoleucine degradation* (Figure S5E and Figure 1I). Increased IFN‐I signalling was not identified in this enrichment analysis, but it was clearly evident in a dedicated differential expression analysis (see Figure S1A for the IFN‐I related differentially expressed genes). The miRNA expression patterns of the iPSC‐derived *ISG15^–/–^* macrophages revealed enrichment of the PI3K‐AKT, FoxO, and MAPK signalling pathways along with various cancer‐related pathways, whereby PI3K‐AKT and FoxO signalling pathways were most closely associated with ISG15 deficiency, as they were enriched in *ISG15^–/–^* cells both with and without IFN‐*α* stimulation (Figure S6D–G).

GSEA of microarray data from the iPSC‐derived ECs revealed interferon‐driven and pro‐inflammatory pathways including *toll‐like receptor signalling, TNF signalling*, and *RIG‐I‐like signalling* among the most significantly enriched in IFN‐α stimulated *ISG15^–/–^* ECs compared to IFN‐α stimulated WT cells, and also some evidence of depletion of energy metabolism and amino acid biosynthesis (Figure S7B).

Taken together, this GSEA suggested that apart from the expected hyperinflammation and IFN‐I signature, increased apoptosis, but decreased PI3K‐AKT signalling, energy generation, nucleotide synthesis, and branched chain amino acid (BCAA) catabolism were cardinal cellular features of ISG15 deficiency and confirmed that a distinct phenotype was apparent without IFN‐α stimulation at least in the *ISG15* ^–/–^ macrophages.

### 3.3 Increased ROS production and reduced AKT phosphorylation modulate GSK3β signalling and apoptosis‐related genes

**FIGURE 2:**
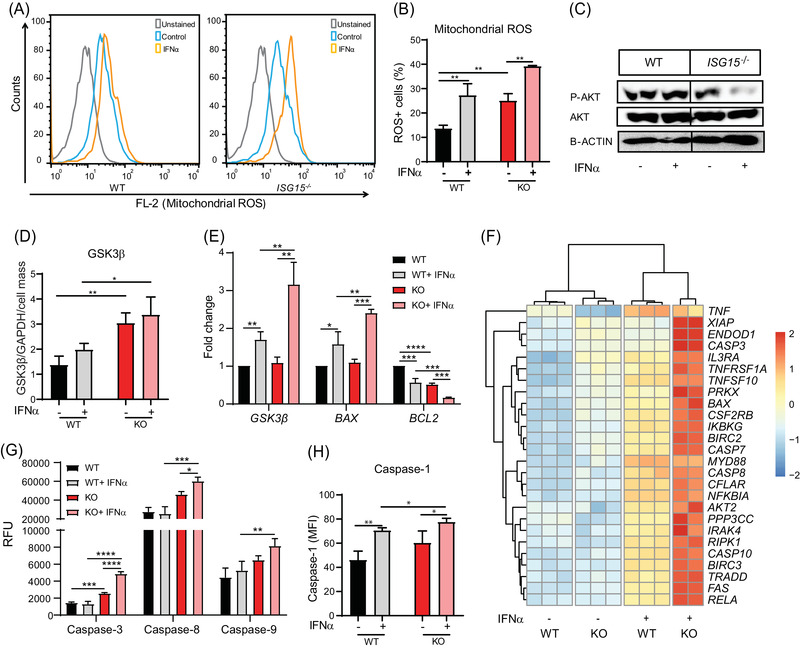
Increased mitochondrial ROS production, reduced AKT phosphorylation, and induction of GSK3β, apoptotic genes, apoptosis, and pyroptosis in *ISG15^–/–^* iPSC‐derived macrophages. WT and *ISG15^–/–^* macrophages were stimulated with 1000 IU/ml of IFN‐α for 24 h as indicated on the *x*‐axes. (A,B) Mitochondrial ROS levels are higher in *ISG15^–/–^* than in WT macrophages with and without IFN‐α stimulation (flow cytometry). (C) Lower levels of phosphorylated (P‐AKT), but not unphosphorylated, AKT in lysates of IFN‐α stimulated *ISG15^–/–^* cells (western blot). (D) Increased levels of GSK3β protein in unstimulated and IFN‐α stimulated *ISG15^–/–^* cells (in‐cell ELISA normalized against GAPDH and crystal violet staining). (E) Increased expression of *GSK3β* mRNA and *BAX* but lower expression of *BCL2* mRNA in IFN‐α stimulated *ISG15^–/–^* cells (RT‐qPCR). (F) Hierarchical clustering analysis based on differentially expressed genes (FDR < .05) as defined by KEGG pathway *apoptosis* (RNA sequencing). (G,H) Increased activity of caspases 3, 8, 9, and 1 in IFN‐α stimulated *ISG15^–/–^* cells: (G) fluorometry (RFU, relative fluorescent units); (H) flow cytometry (MFI, mean fluorescent intensity). *, *p* < .05; **, *p* < .01; ***, *p* < .001; ****, *p* < .0001, one‐way ANOVA followed by Tukey's post hoc test

Considering the increased inflammation and suggestions of mitochondrial dysfunction in *ISG15^–/–^* cells seen in the GSEA, we then measured mitochondrial ROS. Indeed, ROS levels were higher in *ISG15^–/–^* macrophages both before and after IFN‐α stimulation (Figure 2A,B). Since ROS modulate activity of the PI3K‐AKT pathway^39^ and depletion of PI3K activity was seen in the GSEA, we measured AKT phosphorylation (P‐AKT = activated AKT). Indeed, P‐AKT levels were lower in IFN‐α stimulated and, less so, unstimulated *ISG15^–/–^* macrophages (Figure 2C). AKT inactivates glycogen synthase kinase (GSK3β) by phosphorylation.^40^ It was therefore plausible that the decreased AKT phosphorylation activated GSK3β signalling. Indeed, expression of GSK3β mRNA was higher in IFN‐α stimulated *ISG15^–/–^* than WT cells, and GSK3β protein expression was higher in *ISG15^–/–^* cells also without IFN‐α stimulation (Figure 2D,E). GSK3β activity affects expression of pro‐ and anti‐apoptotic genes.^41^ Consistent with the observed increased GSK3β levels, the *ISG15^–/–^* macrophages featured higher transcription of *BAX* (a pro‐apoptotic gene) but lower transcription of the anti‐apoptotic gene *BCL2* (Figure 2E).

### 3.4 Transcriptomic signatures reveal increased oxidative stress and apoptosis in *ISG15^–/–^* macrophages

In order to understand the underlying mechanism(s) of the apparent pro‐apoptotic phenotype of the *ISG15^–/–^* macrophages, we analyzed transcriptomes of the IFN‐α stimulated *ISG15^–/–^* and WT macrophages further. Specifically, *ISG15^–/–^* macrophages exhibited higher expression of *CASP3, CASP7, CASP8, CASP10, BAX, RELA*, and *FAS* mRNA (all of which are functionally related to oxidative stress and apoptosis) as well as, for instance, higher expression of the TNF superfamily and NFκB‐related factors (Figure 2F). We then measured the activity of initiator caspases, CASP‐8 and CASP‐9, and the effector caspase, CASP‐3, via fluorometric assay. Indeed, activity of all three was higher in *ISG15^–/–^* cells, thus confirming their pro‐apoptotic phenotype (Figure 2G).

Expression of CASP1, which induces pyroptotic cell death,^42^, ^43^ was higher in IFN‐α stimulated *ISG15^–/–^* macrophages (Figure 2H). This also explained the higher levels of IL‐1β seen in Figure 1F, as this cytokine is released by cleaved CASP1 and CASP3. Activated CASP1 also cleaves gasdermin‐D (GSDMD), which forms membrane pores resulting in pyroptosis.^44^ Increased expression of *GSDMD* mRNA was evident in *ISG15^–/–^* macrophages (Figure S1B). Consistently, *ISG15^–/–^* cells exhibited elevated CASP1 activity after LPS stimulation (Figure S1F). Together, these findings suggested that increased oxidative stress and propensity for apoptosis and pyroptosis are central features of the cellular phenotype of ISG15 deficiency.

### 3.5 ISG15 deficiency reduces mitochondrial respiration and expression of mitochondrial genes

**FIGURE 3:**
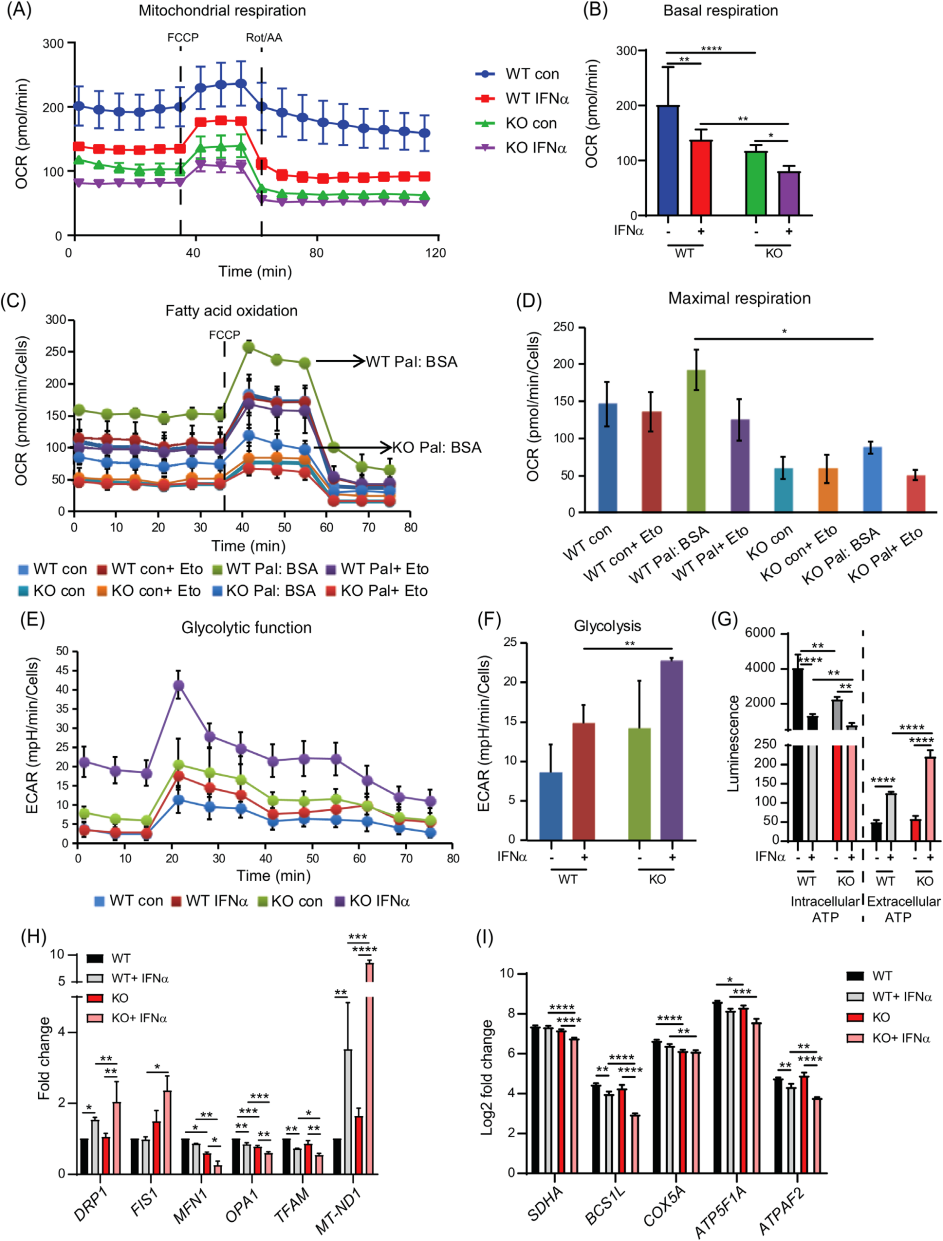
Reduced mitochondrial respiration, fatty acid oxidation (FAO), and increased glycolysis in *ISG15^–/–^* cells. WT and *ISG15^–/–^* macrophages were stimulated with IFN‐α (1000 IU/ml) and oxygen consumption rate (OCR), extracellular acidification rate (ECAR), ATP levels, and expression of selected genes were measured after 24 h. (A,B) OCR is lower in *ISG15^–/–^* than in WT macrophages and decreases in both cell types after IFN‐α stimulation (Seahorse XF Extracellular Flux Analyzer using XF Cell Mito Stress Test). (C,D) Differences in FAO between *ISG15^–/–^* and WT cells (Seahorse XF Extracellular Flux Analyzer, XF Cell Mito Stress Test). The FAO inhibitor etomoxir (Eto) was added to selected groups 15 min prior to starting the XF assay. Palmitate:BSA or Control:BSA was added to selected groups at start of the XF assay to quantify FAO by measuring the resulting change in OCR. OCR was higher in the WT Pal:BSA group than in the KO Pal:BSA group, indicating lower FAO in the *ISG15^–/–^* cells (marked by arrows). (E,F) Higher ECAR in *ISG15^–/–^* than in WT cells after IFN‐α stimulation, indicating higher glycolysis (Seahorse XF Extracellular Flux Analyser, Glycolysis Stress Test). (G) Lower intracellular but higher extracellular ATP levels in IFN‐α stimulated *ISG15^–/–^* cells (luciferase assay). (H) IFN‐α stimulated *ISG15^–/–^* macrophages feature higher expression of mitochondrial fission genes but lower expression of mitochondrial fusion genes (RT‐qPCR). The mitochondrial biogenesis gene *TFAM* is downregulated more in the *ISG15^–/–^* than the WT cell. Expression of the mitochondrial ROS‐responsive gene *Mt‐ND1* (a component of respiratory chain complex I) is markedly higher in IFN‐α stimulated *ISG15^–/–^* than in WT cells. (I) Expression of respiratory chain complex II‐V genes is lower in unstimulated and stimulated *ISG15^–/–^* than WT cells (RNA sequencing). *, *p* < .05; **, *p* < .01; ***, *p* < .001; ****, *p* < .0001, one‐way ANOVA followed by Tukey's post hoc test

The enrichment analysis had suggested reduced *oxidative phosphorylation* and *citrate cycle (TCA cycle)* activity in *ISG15^–/–^* cells (Figure 1I and Figure S5E). Moreover, mitochondrial ROS production may reflect dysfunction of the respiratory chain. We therefore used the Seahorse XF extracellular flux analyser to compare oxidative phosphorylation capacity between *ISG15^–/–^* and WT macrophages. Indeed, mitochondrial respiration was lower in *ISG15^–/–^* than WT macrophages (Figure 3A,B). In agreement with this, intracellular ATP levels were lower in *ISG15^–/–^* cells (Figure 3G). The elevated extracellular ATP levels in *ISG15^–/–^* cells (Figure 3G) were consistent with their propensity for increased apoptosis/pyroptosis shown above (Figure 2 G,H). In addition, fatty acid oxidation was lower in *ISG15^–/–^* macrophages (Figure 3C,D), which agreed well with depletion of *fatty acid oxidation* seen in the GSEA (Figure S5E). We therefore analyzed the acylcarnitine profiles which were available in a targeted metabolomic data set from the WT and *ISG15^–/–^* fibroblasts (Figure S8). Indeed, acylcarnitine profiles in WT and *ISG15^–/–^* cells were distinct. This was mostly due to higher concentrations in the *ISG15^–/–^* cells, indicating a general back‐up of the pathway. In order to assess alternative energy production pathways, we also measured glycolytic activity but found that IFN‐α stimulated *ISG15^–/–^* macrophages featured higher glycolysis (Figure 3E,F). To test whether ISG15 is required for optimal energy metabolism in a different cell type and whether this requires its ISGylation function, we assessed mitochondrial respiration in immortalized *ISG15^–/–^* fibroblasts and in *ISG15^–/–^* fibroblasts stably expressing WT or conjugation‐deficient (ΔGG) ISG15 (Figure S9A,B). As in the iPSC‐derived macrophages, OCR was markedly lower in the *ISG15^–/–^* cells. Of note, transduction of *ISG15^–/–^* cells with the WT allele led to complete rescue of OCR, whereas intermediate OCR was measured in the *ISG15^–/–^* cells expressing the ΔGG mutant. In contrast, expression of the IFN‐regulated mRNAs *ACOD1*, *IFIT1*, and *CXCL10* normalized to the same extent in cells transduced with WT or ΔGG ISG15 (Figure S9C–E), which confirmed previous findings that up‐regulation of these IFN‐driven genes in ISG15 deficiency results from loss of USP18‐mediated inhibition of IFNAR signalling rather than defective ISG15 conjugation.^12^

These results suggest that some, but not all, functions of ISG15 in mitochondrial respiration require its conjugation to cellular targets. In search for potential mechanisms relating to metabolic fluxes, we measured the uptake rate of glucose and glutamine in combination with stable‐isotope‐assisted metabolomics in dermal fibroblasts. Uptake of glutamine (but not glucose) was lower in *ISG15^–/–^* fibroblasts than in WT cells (Figure S10A,B). In line with these results, glutamine contributed less carbon to TCA cycle intermediates in *ISG15^–/–^* fibroblasts than in WT cells, while the contribution from glucose increased (Figure S10C,D). The latter could, however, only partially compensate for the decreased contribution by glutamine, as the absolute increase in carbon contribution from glucose was very small (compare relative carbon contributions in **c** and **d**). Reduced glutamine uptake, leading to lower glutamine oxidation, could therefore contribute to the mitochondrial dysfunction in *ISG15^–/–^* cells. In addition, it may contribute to the reduced *purine* and *pyrimidine metabolism* seen in the GSEA, as glutamine is a precursor of both.

High ROS levels can affect mitochondrial biogenesis, and genes associated with mitochondrial fusion and fission might therefore be affected.^45^ Indeed, expression of the fission‐associated genes *DRP1* and *FIS1* was increased in IFN‐α stimulated *ISG15^–/–^* macrophages compared to the WT cells (Figure 3H). Additionally, the mitochondrial fusion genes *MFN1* and *OPA1* were downregulated in unstimulated and stimulated *ISG15^–/–^* macrophages and there was also reduced expression of *TFAM* in stimulated *ISG15^–/–^* macrophages, one of the most important regulators of mitochondrial biogenesis^46^ (Figure 3H). In addition, expression of the mitochondrial gene *Mt‐ND1*, which encodes a subunit of respiratory chain complex I and is induced in response to elevated ROS,^47^ was higher in *ISG15^–/–^* macrophages after IFN‐α stimulation, thus confirming the innately higher oxidative stress level in these cells. Moreover, expression of nuclear genes encoding components of mitochondrial respiratory chain complexes II‐V was also lower in IFN‐α stimulated *ISG15^–/–^* macrophages, suggesting that the observed defect in mitochondrial respiration may, at least in part, be due to reduced levels of these components (Figure 3I).

Analysis of the macrophage miRNA data further supported the above findings: miR‐98, which has anti‐apoptotic and anti‐oxidative stress functions,^48^ was down‐regulated in IFN‐α stimulated *ISG15^–/–^* macrophages, whereas expression of miR‐302b, which is induced by oxidative stress,^49^ was higher in *ISG15^–/–^* than in WT macrophages (Figure S6B,C).

Taken together, the above results suggest that a heterogeneous mitochondrial dysfunction is a cardinal feature of the cellular phenotype of ISG15 deficiency.

### 3.6 Reduced branched‐chain amino acid amino transferase 1 (BCAT1) expression and branched chain amino acid (BCAA) metabolism in ISG15 deficient cells

**FIGURE 4:**
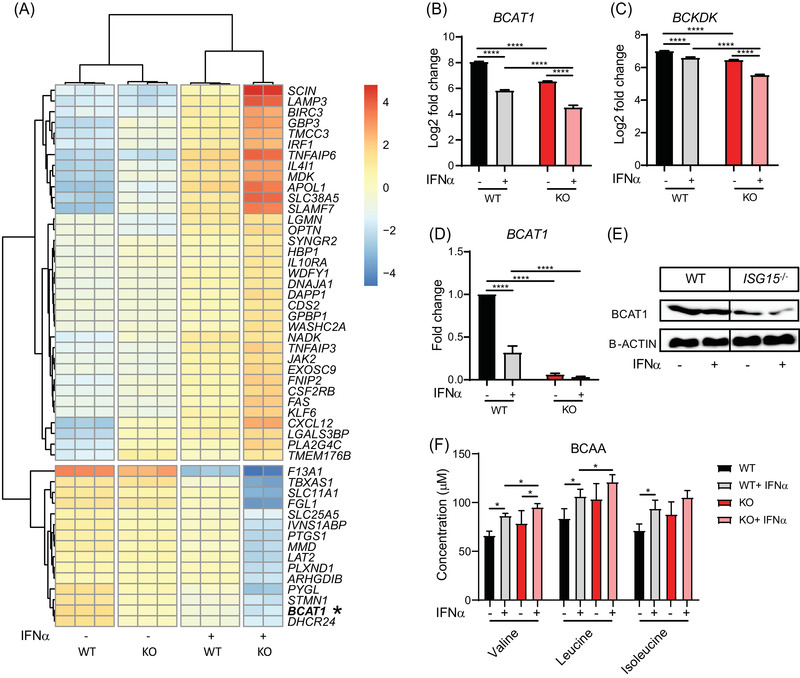
Reduced BCAT1 and BCKDK expression and branched chain amino acid (BCAA) catabolism in *ISG15^–/–^* cells. (A–E) *ISG15^–/–^* and WT macrophages were stimulated with IFN‐α (1000 IU/ml) for 24 h as indicated on the *x‐*axes. (A) Hierarchical biclustering analysis of the 50 most significantly differentially expressed genes across all samples (RNA sequencing). (B,C) Lower expression of *BCAT1* and *BCKDK* mRNA in unstimulated and stimulated *ISG15^–/–^* than WT cells (RNA sequencing). (D,E) Lower expression of *BCAT1* mRNA (RT‐qPCR) and protein (western blot) in stimulated and unstimulated *ISG15^–/–^* than WT cells. (F) BCAA analysis. WT and *ISG15^–/–^* dermal fibroblasts were grown in the presence or absence of IFN‐α (1000 IU/ml), and concentrations of the indicated BCAA were measured by mass spectrometry after 24 h. Valine and leucine concentrations are significantly higher in IFN‐α stimulated *ISG15^–/–^* than WT cells, and there is a tendency for increase in isoleucine. *, *p* < .05; **, *p* < .01; ***, *p* < .001; ****, *p* < .0001, one‐way ANOVA followed by Tukey's post hoc test

BCAT1 is responsible for catabolism of BCAA, which results in formation of branched chain α‐ketoacids (BCKA) and glutamate.^50^ After transamination, BCKA are converted to acetyl‐CoA, which is later oxidized in the TCA cycle. It has been reported that higher BCAT1 expression correlates with reduced oxidative stress.^51^ Indeed, the macrophage RNAseq data revealed reduced levels of *BCAT1* mRNA (Figure 4A,B) and also of mRNA encoding branched‐chain α‐ketoacid dehydrogenase kinase (*BCKDK*), another key enzyme in BCAA catabolism^52^ (Figure 4C). We verified mRNA and protein expression of BCAT1 in an independent experiment and, indeed, its expression was markedly reduced in *ISG15^–/–^* macrophages (Figure 4D,E). Analysis of amino acid concentrations in the targeted metabolomics data set from *ISG15^–/–^* and WT dermal fibroblasts indicated a tendency toward lower BCAA catabolism, as reflected by higher concentrations of valine, leucine, and isoleucine in *ISG15^–/–^* cells (Figure 4F). These findings suggest that a relative BCAT1 deficiency contributes to the observed reduced mitochondrial respiration in *ISG15^–/–^* cells after IFN‐α stimulation.

### 3.7 Further validation of the *ISG15^–/–^* phenotype by analysis of gene expression in iPSC‐derived EC

The phenotypic features of iPSC‐derived macrophages were further verified by microarray analysis of iPSC‐derived EC. As in the macrophages, the greatest degree of transcriptional change (compared to unstimulated WT cells) was detected in IFN‐α stimulated *ISG15^–/–^* cells (Figure S11A–C). Specifically, there was upregulation of genes related to type I IFN signature (e.g., *IFIT2, MX2, IFI27* and *ISG20*), hyperinflammation (e.g., *TLR3*, *CXCL10* and *CXCL11*) (Figure S11D), and downregulation of genes related to PI3K‐AKT signalling (*AKT1*) and ROS scavenging (*HMOX1*, *NQO1*) (Figure S11E) in IFN‐α stimulated *ISG15^–/–^* cells as compared to WT cells. Additionally, there was higher expression of apoptosis associated genes (*CASP9, CASP1, CASP3, CASP7, CASP10*, *CASP8*, *TNFSF10*, and *FAS*) and the pyroptosis mediator *GSDMD* (Figure S11D,F), but reduced expression of mitochondrial biogenesis genes (*MFN1*, *OPA1*) and *BCAT1* in IFN‐α stimulated *ISG15^–/–^* EC as compared to WT cells (Figure S11E). These results underscored the importance of ISG15 to regulate oxidative stress and apoptosis in different cell types and underscored the shared features of ISG15 deficiency between macrophages and EC.

### 3.8 IA and derivatives reduce type I IFN signature, hyperinflammation, and ROS production

**FIGURE 5:**
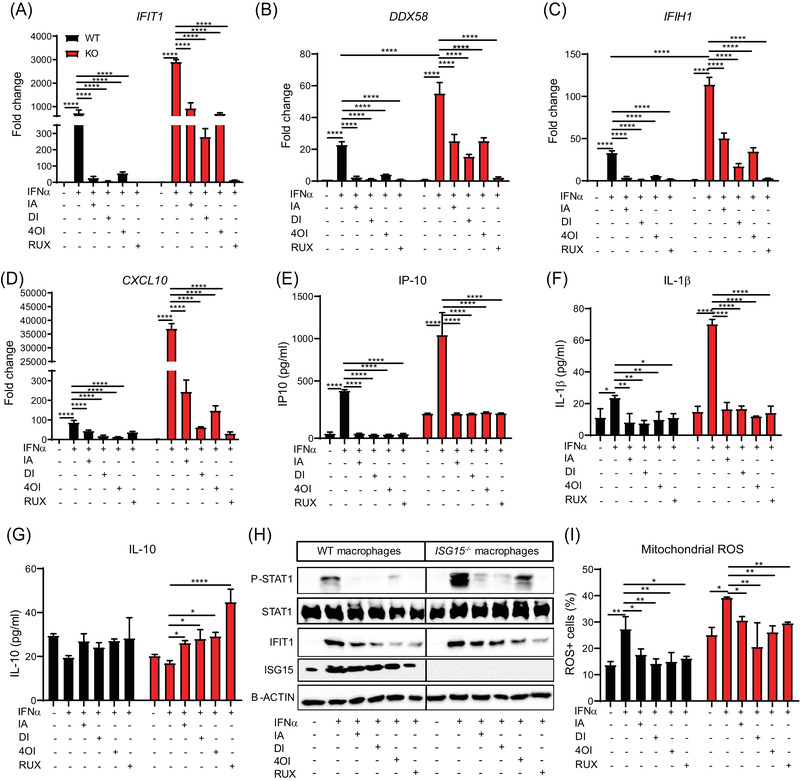
Treatment with itaconate and derivatives reduces type I IFN signature, hyperinflammation, and ROS production in *ISG15^–/–^* iPSC‐derived macrophages. WT and *ISG15^–/–^* macrophages were stimulated with IFN‐α (1000 IU/ml) for 8 h and treated with itaconate (IA, 10 mM), DI (0.5 mM), 4OI (25 µM) and RUX (1 µM) for 16 h. (A–G) Treatment‐induced reduction of the indicated ISGs and proinflammatory cytokines, but enhancement of IL‐10 levels: (A–D) RT‐qPCR; (E–G) ELISA of cell culture supernatants. (H) Treatment‐induced reduction of phosphorylated STAT1 (P‐STAT1) and IFIT1 expression in IFN‐α stimulated *ISG15^–/–^* and WT macrophages (western blot of cell lysates). The blot also demonstrates the absence of ISG15 protein in the *ISG15^–/–^* cells. (I) Treatment‐induced reduction of mitochondrial ROS^+^ cells (flow cytometry). *, *p* < .05; **, *p* < .01; ***, *p* < .001; ****, *p* < .0001, one‐way ANOVA followed by Tukey's post hoc test

In order to evaluate IA and derivatives as treatments for the dysfunctional phenotype of *ISG15^–/–^* cells, we determined nontoxic doses of IA (10 mM) and DI (.5 mM) (Figure S12A,B), while using concentrations of 4OI (25 µM) and RUX (1 µM) that are generally known to be nontoxic. RUX is a well‐characterized JAK1/2 inhibitor that reduces type I IFN responses^53^ and we used it as positive control and comparator for the relative effectiveness of the IA compounds. Of note, when these concentrations were verified to be nontoxic in the MTT assay, less formazan precipitate formation (indicating lower NAD(P)H‐dependent oxidoreductase activity) was obtained with untreated *ISG15^–/–^* than with untreated WT macrophages, which was reduced further by IFN‐α (Figure S12C). This suggested that loss of ISG15 compromises not only mitochondrial respiration (see Figure 3A) but also extramitochondrial NAD(P)H availability. The latter may be in part due to depressed synthesis of its adenine component, as suggested by depletion of *purine synthesis* in the GSEA of WT and *ISG15^–/–^* macrophages shown in Figure S5A, and it may also relate to depletion of *pyruvate metabolism* (in which NADP+ is a cofactor) in the same analysis. Of note, the treatments brought the MTT signal of IFN‐α stimulated *ISG15^–/–^* cells up to the level of unstimulated *ISG15^–/–^* cells, but not up to the level of WT cells. In contrast, when unstimulated *ISG15^–/–^* macrophages were treated, the MTT signal increased to near WT levels, indicating that the compounds could rectify oxidoreductase activity in these cells when there was no additional IFN‐signalling (Figure S12D). These non‐toxic doses were then used to test effects of the compounds on hyperinflammation in IFN‐α stimulated *ISG15^–/–^* macrophages. Treatment with all four compounds led to significantly reduced expression of *IFIT1, DDX58, IFIH1*, and *CXCL10* mRNA and IP‐10 and IL‐1β protein (Figure 5A–F), but to increased levels of the predominantly anti‐inflammatory IL‐10 (Figure 5G). Likewise, all four compounds reduced the expression of P‐STAT1 and IFIT1 (Figure 5H). A normalization of IFN‐I responses by IA and 4OI was also verified in single‐cell transcriptomes (Figure S13A–C). There was a comparable reduction of IFN‐I responses and hyperinflammation in ECs (Figure S13D–H), HaCaT cells (Figure S14A–E) and fibroblasts (Figure S14F–I). The treatment effect was also evident in LPS‐stimulated macrophages, but RUX was not as effective as the itaconates, which is consistent with the lesser induction of IFN‐I signalling by LPS than by IFN‐α (Figure S15). Treatment with all four compounds also reduced mitochondrial ROS production in *ISG15^–/–^* macrophages to a comparable extent (Figure 5I).

### 3.9 IA and derivatives induce AKT phosphorylation and reduce GSK3β expression

**FIGURE 6:**
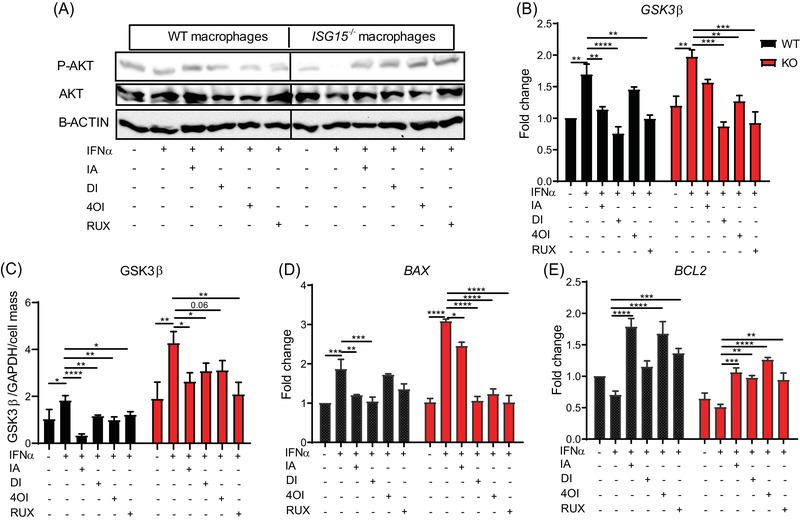
Treatment with itaconate and derivatives induces AKT phosphorylation and reduces GSK3β signalling and apoptotic genes in *ISG15^–/–^* macrophages. WT and *ISG15^–/–^* macrophages were stimulated with IFN‐α (1000 IU/ml) for 8 h and then treated with itaconate (IA, 10 mM), DI (0.5  mM), 4OI (25 µM), and RUX (1 µM) for 16 h. (A–E) The treatments increase phosphorylated AKT levels in IFN‐α stimulated *ISG15^–/–^* cells, but decrease *GSK3β* mRNA and protein levels, as well as *BAX* mRNA, whereas *BCL2* mRNA expression increases upon treatment: (A) western blot of cell lysates; (B,D,E) RT‐qPCR; (C) ELISA of cell lysates normalized to GAPDH and cell number as determined by crystal violet staining. *, *p* < .05; **, *p* < .01; ***, *p* < .001; ****, *p* < .0001, one‐way ANOVA followed by Tukey's post hoc test

The results shown in Figure 2C had suggested reduced AKT phosphorylation in *ISG15^–/–^* macrophages, which correlated with increased ROS levels. Consistent with their anti‐ROS activity shown above, the treatments increased AKT phosphorylation in the *ISG15^–/–^* macrophages (Figure 6A). Likewise, they reduced levels of *GSK3*β mRNA and protein (Figure 6B,C) and *BAX* mRNA expression, but increased *BCL2* expression (Figure 6D,E). Thus, IA and derivatives reprogram the AKT/GSK3 signalling pathway to reduce levels of pro‐apoptotic mediators.

### 3.10 IA and derivatives reduce apoptosis and pyroptosis

**FIGURE 7:**
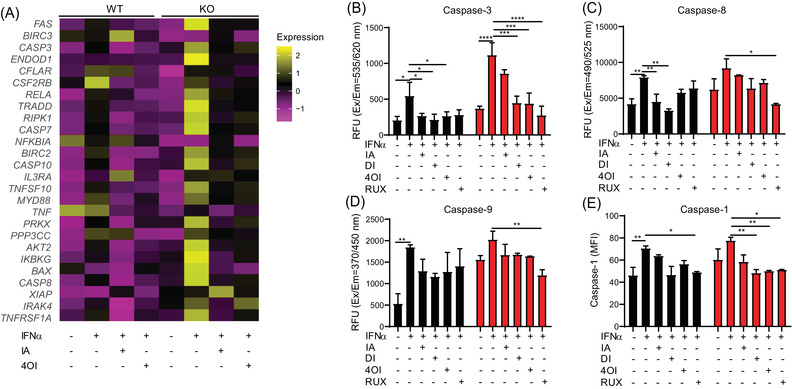
Reduction of apoptosis and pyroptosis in *ISG15^–/–^* iPSC‐derived macrophages treated with itaconate and derivatives. WT and *ISG15^–/–^* macrophages were stimulated with IFN‐α (1000 IU/ml) for 8 h and treated with itaconate (IA, 10 mM), DI (0.5 mM), 4OI (25 µM) and RUX (1 µM) for 16 h. (A) The heatmap shows relative expression of differentially expressed (FDR < .05) genes in the KEGG pathway *apoptosis* (single cell‐RNA sequencing). The pro‐apoptotic signature of the *ISG15^–/–^* cells is effectively reduced by treatment with itaconate and 4OI. (B–E) Reduction of caspase 3, 8, 9, and 1 activity by the treatments: (B–D) fluorometry (RFU, relative fluorescent units); (E) flow cytometry (MFI, mean fluorescent intensity). *, *p* < .05; **, *p* < .01; ***, *p* < .001; ****, *p* < .0001, one‐way ANOVA followed by Tukey's post hoc test

Considering the downregulation of *GSK3*β and *BAX* expression in *ISG15^–/–^* macrophages treated with IA and derivatives, we analyzed the single cell‐RNA sequencing data of iPSC‐derived macrophages under treatment with the four compounds for expression of genes involved in apoptosis. In agreement with the bulk RNAseq data (Figure 2F) and microarray analysis of iPSC‐derived EC (Figure S11F), there was strong upregulation of, for example, *CASP3, CASP7, CASP8, CASP10, FAS, BAX, RELA* and *TNFSF10* in *ISG15^–/–^* macrophages stimulated with IFN‐α, which was effectively reversed by the treatments (Figure 7A). Additionally, we found in the microarray data of ECs that the pro‐apoptotic differentially expressed genes (*CASP9, CASP1, CASP3, CASP7, CASP10* and *CASP8*) were upregulated by IFN‐α in *ISG15^–/–^* cells, but were effectively downregulated by the treatments (Figure S11F). To confirm these findings, we also measured caspase 3, 8, and 9 activity. Indeed, caspase 3, 8, and 9 activity increased in *ISG15^–/–^* macrophages after stimulation with IFN‐α, which was reduced by the treatments (Figure 7B–D). Moreover, increased caspase 1 activity in *ISG15^–/–^* macrophages stimulated with IFN‐α was reduced by treatment with the itaconates (Figure 7E). Consistent with this, the microarray data of ECs revealed that the treatments could at least partially reverse the strong induction of *GSDMD* in *ISG15^–/–^* ECs (Figure S11D).

### 3.11 Impact of itaconates on the dysfunctional mitochondrial phenotype

**FIGURE 8:**
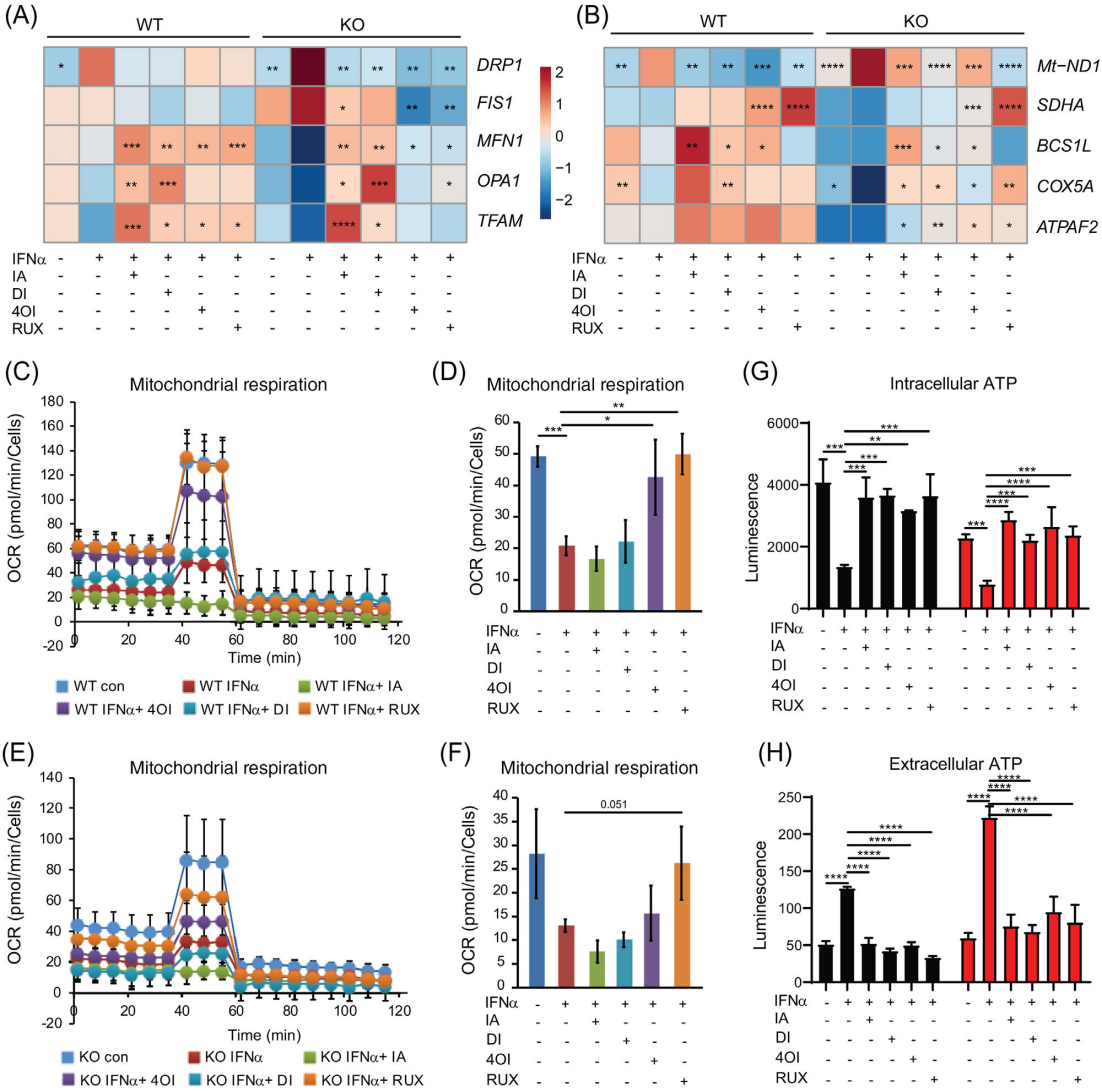
Rescue of the mitochondrial phenotype by treatment with itaconates and RUX. WT and *ISG15^–/–^* iPSC‐derived macrophages were stimulated with IFN‐α (1000 IU/ml) for 8 h and treated with itaconate (IA, 10 mM), DI (0.5  mM), and 4OI (25 µM) for 16 h. (A) Expression changes of mitochondrial biogenesis genes upon treatment (RT‐qPCR). Treatments reduce expression of mitochondrial fission genes *DRP1* and *FIS1*, increase expression of fusion genes *MFN1* and *OPA1* and mitochondrial biogenesis gene *TFAM*. (B) Expression changes of respiratory chain complex I–V genes upon treatment (RT‐qPCR). Treatments decrease expression of ROS‐responsive gene *Mt‐ND1* (component of complex I) but increase expression of respiratory chain complex II–V genes. Statistical significance was determined with respect to the untreated, IFN‐α stimulated WT or *ISG15^–/–^* group. (C–F) OCR is restored by treatment with RUX and 4OI in IFN‐α stimulated WT cells (C,D), whereas partial rescue of OCR is observed by treatment with RUX and 4OI in IFN‐α stimulated *ISG15^–/–^* cells (E,F) (Seahorse XF Extracellular Flux Analyzer, XF Cell Mito Stress Test). Seahorse XF extracellular flux analyser using XF cell mito stress test) (G,H) Treatment with all four compounds rectifies abnormal intra‐ and extracellular ATP levels in IFN‐α stimulated *ISG15^–/–^* cells (luciferase assay). *, *p* < .05; **, *p* < .01; ***, *p* < .001; ****, *p* < .0001, one‐way ANOVA followed by Tukey's post hoc test

We had observed lower mitochondrial respiration in *ISG15^–/–^* than in WT macrophages (Figure 3A), but only a partial rescue of cellular oxidoreductase activity (MTT assay) by all 4 compounds (Figure S12C,D). Nonetheless, the compounds reduced expression of the mitochondrial fission genes *DRP1* and *FIS1* and increased expression of the fusion genes *MFN1* and *OPA1* (Figure 8A). Also, the treatments increased relative expression of *TFAM* but they decreased *Mt‐ND1* expression (Figure 8A,B). In addition, expression of the mitochondrial biogenesis genes *MFN1* and *OPA1* was reduced in *ISG15^–/–^* ECs stimulated with IFN‐α, which was reversed by the treatments (Figure S11E). Interrogation of the bulk RNAseq data had shown that expression of 5 genes encoding respiratory electron chain components was reduced in *ISG15^–/–^* macrophages (Figure 3I). We measured their expression by RT‐qPCR in an independent treatment experiment (Figure 8B). This verified their downregulation in *ISG15^–/–^* macrophages and revealed that itaconate treatments increased their expression. The catalytic subunit of SDH (SDHA), of which itaconate is a competitive inhibitor,^54^ formed a notable exception in that IA and DI did not affect its expression significantly. We also observed rescue of mitochondrial respiration by RUX and 4OI in WT cells but only partial rescue in *ISG15^–/–^* cells (Figure 8C–F). Of note, only 4OI and RUX corrected the defect in mitochondrial respiration, whereas addition of IA even led to a complete loss of reserve respiratory capacity in WT and *ISG15^–/–^* cells (Figure 8C–F; green lines in C and E), likely due to the additional inhibition of SDH activity. Nonetheless, all four compounds corrected the reduced intracellular and elevated extracellular ATP levels seen in *ISG15^–/–^* cells, demonstrating that their positive impact on global cell energy balance is substantial (Figure 8G,H). Together with the results of the MTT assay (Figure S12C,D), these results suggest that stimulation of mitochondrial biogenesis and improved mitochondrial and cytosolic energy homeostasis are possible mechanisms by which itaconates and RUX provide at least a partial rescue of cell respiration.

### 3.12 Treatment with itaconates enhances BCAT1 expression and BCAA catabolism and improves cellular redox balance in *ISG15^–/–^* cells

**FIGURE 9:**
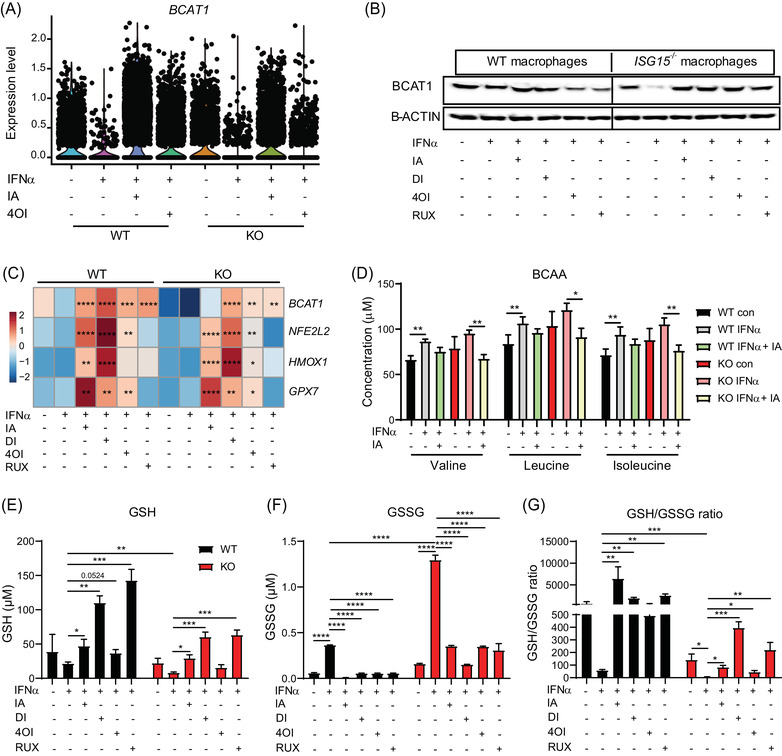
Itaconates and RUX rescue BCAT1 expression and redox balance in IFN‐α stimulated *ISG15^–/–^* cells, but only itaconates induce expression of anti‐oxidant genes *NFE2L2*, *HMOX1*, and *GPX7*. (A–E) WT and *ISG15^–/–^* iPSC‐derived macrophages were stimulated with IFN‐α (1000 IU/ml) for 8 h and treated with itaconate (IA, 10 mM), DI (0.5  mM), 4OI (25 µM), and RUX (1 µM) for 16 h. (A,B) Treatments rescue IFN‐α induced downregulation of *BCAT1* mRNA (scRNAseq, each dot represents one cell) and BCAT1 protein (western blot of cell lysates). (C) Itaconates and RUX upregulate *BCAT1* mRNA in IFN‐α stimulated WT and *ISG15^–/–^* cells, but only itaconates increase expression of anti‐oxidant genes *NFE2L2*, *HMOX1*, and *GPX7* (RT‐qPCR). Statistical significance was determined with respect to the untreated, IFN‐α stimulated WT or *ISG15^–/–^* group. (D) WT and *ISG15^–/–^* dermal fibroblasts were stimulated with IFN‐α (1000 IU/ml) for 8 h and treated with itaconate (25 mM) for 16 h. Concentrations of BCAA valine, leucine, and isoleucine were measured by mass spectrometry. Itaconate treatment reduces levels of all three in IFN‐α treated *ISG15^–/–^* cells, indicating increased BCAT1 activity. (E–G) WT and *ISG15^–/–^* iPSC‐derived macrophages were stimulated with IFN‐α (1000 IU/ml) for 8 h and treated with itaconate (IA, 10 mM), DI (0.5 mM), 4OI (25 µM), and RUX (1 µM) for 16 h. GSH (reduced glutathione) and GSSG (oxidized glutathione) concentrations were measured with a fluorescence microplate reader (Ex/Em = 490/520 nm). Treatments increase GSH and decreased GSSG concentrations, resulting in improved redox balance (higher GSH/GSSG ratio). *, *p* < .05; **, *p* < .01; ***, *p* < .001; ****, *p* < .0001, one‐way ANOVA followed by Tukey's post hoc test

We had observed reduced BCAT1 expression and BCAA catabolism in *ISG15^–/–^* macrophages (Figure 4A,B and Figure S5F). The treatments increased *BCAT1* expression, which was seen in the single‐cell RNAseq data of macrophages, by immunoblot, and in a dedicated RT‐qPCR analysis (Figure 9A–C). Analysis of the microarray data of WT and *ISG15^–/–^* ECs also confirmed that the treatments enhanced *BCAT1* expression, albeit less than in macrophages (Figure S11E). To verify the functional relevance of IA treatment for BCAT1 activity, we analyzed BCAA concentrations in the targeted metabolomics data set from WT and *ISG15^–/–^* dermal fibroblasts. Indeed, IA treatment resulted in significantly reduced concentrations of the three BCAA in IFN‐α stimulated *ISG15^–/–^* cells, suggesting increased catabolism (Figure 9D).

BCAT1 also induces the NRF2 pathway and expression of ROS scavengers.^51^ In agreement with this, itaconate treatments increased expression of *NFE2L2* (encoding NRF2), and the NRF2 target genes *HMOX1* and *GPX7* (Figure 9C). However, this effect was more likely due to direct activation of NRF2 signalling by the itaconates, as RUX (which increased BCAT1 mRNA and protein levels but is not a direct NRF2 agonist) did not affect expression of these mRNAs. To assess the impact of ISG15 deficiency and the treatments on net redox state of the cells, we measured reduced (GSH) and disulfide (GSSG) glutathione as bioindicators (Figure 9E–G). Indeed, GSH/GSSG ratio (indicating a favourable redox balance) was significantly lower in the *ISG15^–/–^* cells, but increased under treatment with all four compounds, with DI exerting the strongest effect, followed by RUX. These findings support the hypothesis that treatment of ISG15‐deficient cells with itaconates improves cellular redox balance by mechanisms involving NRF2 signalling and BCAT1 activity.

### 3.13 Reduction of IFN expression by treatment with IA does not lead to increased viral replication

Patients with ISG15 deficiency do not have an increased risk of viral infections, and *ISG15^–/–^* cells are less susceptible to certain viral infections, possibly due to the persistently elevated IFN levels.^14^ We tested whether treatment with the compounds would raise cellular susceptibility to influenza A virus, one of the most important respiratory pathogens in all age groups. As expected, viral infectivity was lower in *ISG15^–/–^* than in WT macrophages. Remarkably, despite a striking reduction of the excess induction of ISGs in the infected *ISG15^–/–^* cells by treatment with both IA and DI, viral infectivity remained reduced (Figure S16).

## 4 DISCUSSION

Using a variety of human cells types and analytical techniques, we identified an increased propensity for cell death coupled with major defects in mitochondrial homeostasis, energy generation, and redox balance as cardinal features of the cellular phenotype of ISG15 deficiency. Moreover, we identified itaconates and the JAK1/2 inhibitor RUX as effective treatments for most aspects of this multifaceted phenotype.

The fact that the conjugation‐deficient mutant (ΔGG) only partially restored mitochondrial respiration in *ISG15^–/–^* cells suggests that ISG15 participates in a complex regulatory network governing cell respiration in which some of its functions require conjugation to target proteins. Indeed, ISG15‐conjugated proteins in murine and human cells include several enzymes involved in carbohydrate energy metabolism.^55^ Our observation of increased glycolysis in *ISG15^–/–^* cells agrees well with recent findings by Yan et al. that ISG15 limits glycolysis by covalent binding to glycolytic enzymes.^56^ A functional connection between ISG15 and branched chain amino acid catabolism has not been described. BCAT1 is the key enzyme responsible for conversion of branched chain amino acids toward branched chain keto acids and the formation of macromolecular precursors.^57^ These authors also showed that BCAT1 has additional “moonlighting” functions in that it contributes to efficient function of the TCA cycle by maintaining levels of α‐ketoglutarate and 2‐OH‐glutarate. In addition, BCAT1 induces ROS scavengers and attenuates ROS accumulation,^51^ and its expression in tumour cells is associated with enhanced proliferation and decreased apoptosis. Together with these studies, our findings suggest a central role for a relative BCAT1 deficiency in the full manifestation of the cellular phenotype of ISG15 deficiency and it may also play a role in the decreased apoptosis observed in *ISG15^–/–^* cells, as BCAT1 is generally thought to have anti‐ROS and anti‐apoptotic effects.^51^

Work on bone‐marrow derived macrophages from *ISG15^–/–^* mice showed that ROS levels were actually lower in cells from KO mice during infection with vaccinia virus.^19^ ROS levels, as a physiological by‐product of oxidative phosphorylation, usually drop along with mitochondrial respiration. Our results suggest the polar opposite, and it is conceivable that this may be yet another scenario where ISG15 has different effects in humans than in mice. The underlying mechanism of the increased ROS levels is not clear at this time. We favour a combination of decreased ROS scavengers (due to compromised BCAT1/NRF2 activity) and increased IFN levels, as it is well known that the latter raise intracellular ROS levels. In addition, they likely relate to an underlying mitochondrial dysfunction, as ISG15 interacts directly with mitochondrial proteins responsible for autophagy, mitochondrial function, and apoptosis.^13^, ^19^, ^58^ Furthermore, expression of genes supporting mitochondrial biogenesis and encoding respiratory chain complexes II‐V was diminished in ISG15‐deficient cells. Also considering the propensity for apoptosis in *ISG15^–/–^* cells, it is plausible that release of DNA from dysfunctional mitochondria into the cytosol contributes to the hyperinflammation of ISG15 deficiency. Even though not investigated in detail, our data also suggested increased autophagy and mitophagy due to loss of *ISG15*. Taken together, our results therefore suggest mitochondrial dysfunction of diverse causes and consequences as a cardinal, previously unrecognized feature of human ISG15 deficiency. Its clinical significance is uncertain at this time and will require detailed prospective investigations of affected kindreds. In analogy to other associations between oxidative stress and mitochondrial dysfunction and epilepsy,^59^ it is tempting to speculate that the mitochondrial pathology may be one factor contributing to the high risk of seizures in these children.

Overall, itaconates and RUX had similar beneficial effects on nearly all aspects of this multifaceted phenotype of ISG15 deficiency, suggesting that they either acted through their previously documented anti‐IFN effects^24^, ^25^ or affected the same processes by different mechanisms. The latter may be the case with pyroptosis, as IA has been shown to inhibit it by directly interacting with NLRP3 and thus interfering with inflammasome activation.^60^ Interestingly, itaconates are well‐documented activators of the anti‐oxidative NRF2 signalling pathway^24^ and were significantly more effective than RUX in inducing NRF2 target genes, the ROS scavengers *HMOX1* and *GPX7*.^61^ Irrespective of the observed differences between itaconates and RUX, or even among the itaconates themselves, it should be noted that all four compounds greatly improved overall cell energy state (intracellular ATP) and redox balance (GSH/GSSG ratio), and normalized all parameters of hyperinflammation. Taken together, our data therefore suggest that IA‐derived treatments may be at least equivalent to RUX, and that type I interferonopathies should be considered as one disease indication as therapeutics based on IA progress toward clinical applications. Notably, treatment with IA and DI reduced IFN‐I levels but did not alter the reduced infectivity of *ISG15^–/–^* macrophages by influenza A virus. It is conceivable that the increased IFN‐signalling alone accounts for the reduced susceptibility of *ISG15*‐deficient cells for certain viruses. However, our results suggest strongly that this is not the case, but that other ISG15‐regulated mechanisms are at work. IA has antiviral properties^62^ and one might therefore argue that this masked an increased viral infectivity that would be expected under normalized IFN levels. However, we can exclude this possibility because neither IA nor DI affect HA mRNA levels in human macrophages.^25^ From a clinical perspective it appears remarkable that, inferring from this reductionist model, there is no evidence to suspect that targeted treatment of ISG15 deficiency would increase the risk of viral infections of the patients.

Taken together, our results suggest (i) that ISG15 deficiency leads to a complex cellular phenotype that extends beyond the well‐described hyperinflammatory manifestations to encompass a mitochondrial dysfunction of potentially broad consequences, and (ii) that pharmacological agents based on IA merit further investigation as alternate treatment options.

## CONFLICT OF INTEREST

The authors declare that there is no conflict of interest that could be perceived as prejudicing the impartiality of the research reported.

## Supporting information

Figure S1. Hyperinflammatory phenotype of *ISG15^‐/‐^* hiPSC‐derived macrophages

Figure S2. IFN‐α inducible hyperinflammatory phenotype of *ISG15^‐/‐^* hiPSC‐derived ECs

Figure S3. IFN‐α inducible hyperinflammatory phenotype of *ISG15^‐/‐^* HaCaT keratinocytes

Figure S4. IFN‐α inducible hyperinflammatory phenotype of *ISG15^‐/‐^* dermal fibroblasts

Figure S5. IFN‐α stimulation effects major changes in transcriptomes in WT and *ISG15^‐/‐^* iPSC‐derived macrophages.

Figure S6. miRNA populations in WT and *ISG15^‐/‐^* iPSC‐derived macrophages differ according to genotype and IFN‐α stimulation

Figure S7. Differences in transcriptomes due to IFN−α stimulation of iPSC‐derived WT and *ISG15^‐/‐^* ECs

Figure S8. Differences in acylcarnitine populations in WT and *ISG15^‐/‐^* fibroblasts support the existence of a global defect in energy metabolism in *ISG15^‐/‐^* cells

Figure S9. Full restoration of mitochondrial respiration in *ISG15^‐/‐^* fibroblasts requires the ISGylation ability of ISG15

Figure S10. Stable‐isotope assisted metabolic flux studies of WT and *ISG15^‐/‐^* dermal fibroblasts reveal decreased glutamine uptake and glutamine‐derived carbon contribution to the TCA cycle

Figure S11. Microarray analysis reveals differential impact of itaconate treatments on gene expression in WT and *ISG15^‐/‐^* iPSC‐derived ECs

Figure S12. A,B. Determination of nontoxic doses of IA and DI in IFN‐α stimulated immortalized dermal fibroblasts for treatment with itaconate and derivatives. C,D. Effects of itaconates and RUX on NAD(P)H‐dependent oxidoreductase activity in unstimulated and stimulated WT and *ISG15^‐/‐^* macrophages

Figure S13. A–C. Single‐cell RNAseq analysis of iPSC‐derived macrophages. D‐H. Treatment with itaconate and derivatives reduces type I IFN signature and hyperinflammation in iPSC‐derived *ISG15^‐/‐^* ECs.

Figure S14. Itaconate and derivatives reduce type I IFN signature and hyperinflammation in *ISG15^‐/‐^* HaCaT cells and dermal fibroblasts

Figure S15. Treatment with itaconate and derivatives reduces type I IFN signature and hyperinflammation in LPS‐stimulated iPSC‐derived *ISG15^‐/‐^* macrophages

Figure S16. Treatment with itaconate and DI reverts hyperinflammation in influenza A virus infected *ISG15^‐/‐^* iPSC‐derived macrophages but does not increase viral infectivity

Figure S17. Basic quality data of single‐cell RNAseq analysis of iPSC‐derived macrophages

Table S1. List of RT‐qPCR primers
