# Supplementary figures and images for: ISG15 deficiency features a complex cellular phenotype that responds to treatment with itaconate and derivatives
Source: Clin Transl Med. 2022 Jul 17;12(7):e931. doi: 10.1002/ctm2.931 (PMC9288839; doi:10.1002/ctm2.931)

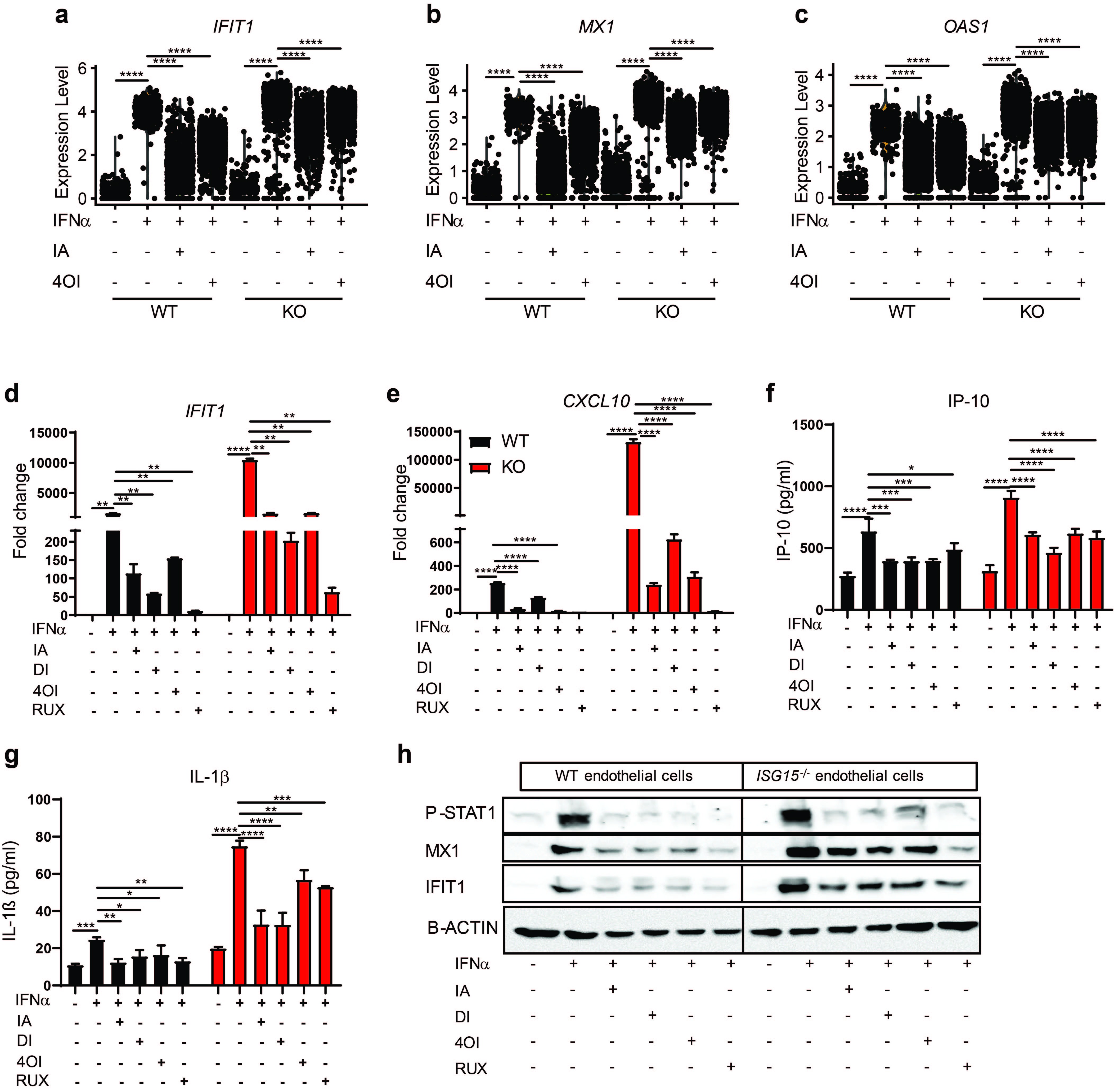

Supplement: Supplementary file 13 — Figure S13. A–C. Single‐cell RNAseq analysis of iPSC‐derived macrophages. D‐H. Treatment with itaconate and derivatives reduces type I IFN signature and hyperinflammation in iPSC‐derived ISG15‐/‐ ECs. [file CTM2-12-e931-s012.jpg]

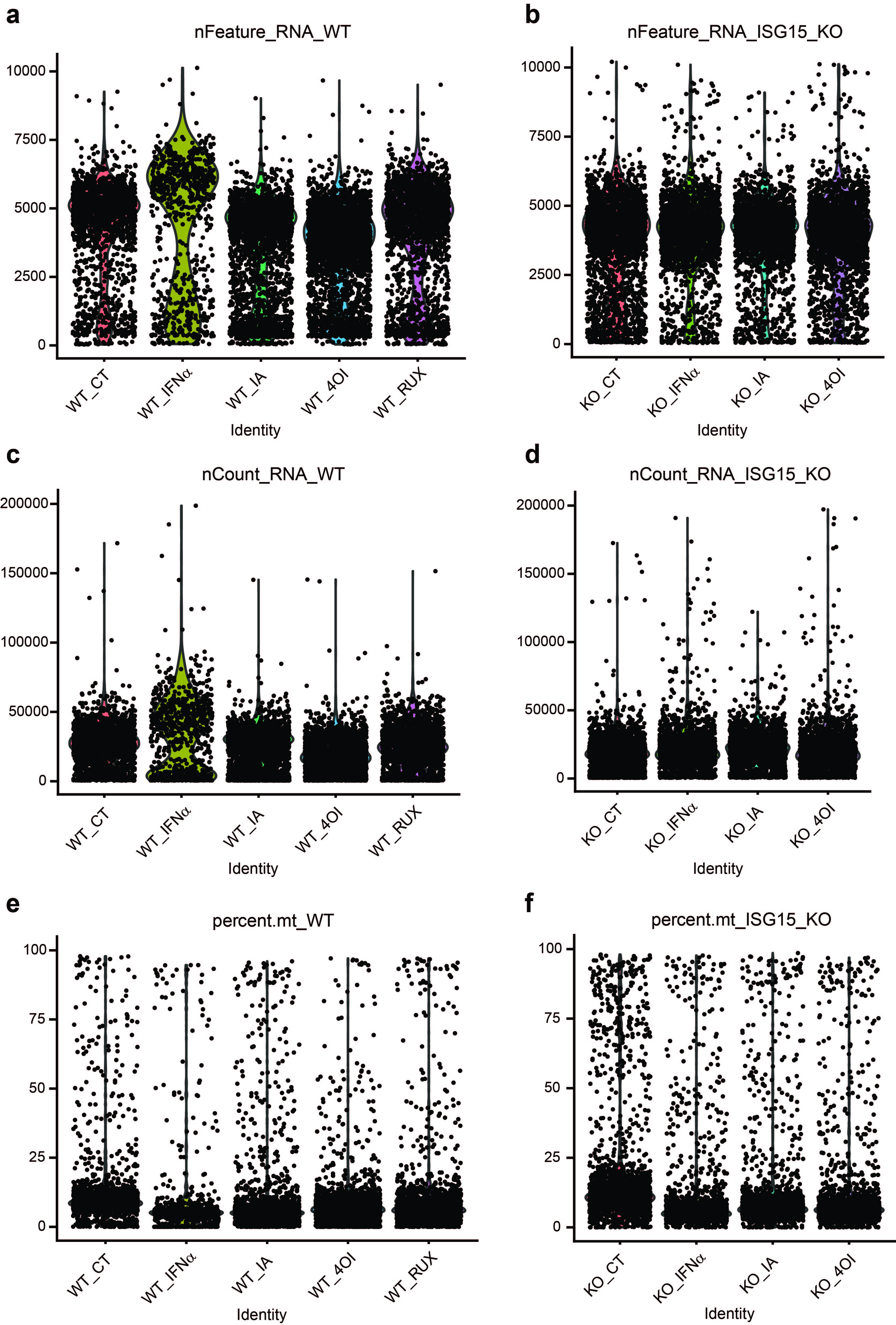

Supplement: Supplementary file 17 — Figure S17. Basic quality data of single‐cell RNAseq analysis of iPSC‐derived macrophages [file CTM2-12-e931-s007.jpg]
