# Supplementary material for: ISG15 deficiency features a complex cellular phenotype that responds to treatment with itaconate and derivatives
Source: Clin Transl Med. 2022 Jul 17;12(7):e931. doi: 10.1002/ctm2.931 (PMC9288839; doi:10.1002/ctm2.931)
Supplement: Supplementary file 18 — Table S1. List of RT‐qPCR primers [file CTM2-12-e931-s003.doc]

| **Supplementary table 1. List of RT-qPCR primers** | | |
| --- | --- | --- |
| **Gene name** | **Primer name** | **Sequence (5´ - 3´)** |
| ***ISG15*** | ISG15-F | CAGCGAACTCATCTTTGCCAG |
|  | ISG15-R | GACACCTGGAATTCGTTGCC |
| ***IFIT1*** | IFIT1-F | TCAGGCATTTCATCGTCATC |
|  | IFIT1-R | GCAGAACGGCTGCCTAATTT |
| ***OAS1*** | OAS1-F | TGACTGGCGGCTATAAACC |
|  | OAS1-R | TGGGCTGTGTTGAAATGTGT |
| ***MX1*** | MX1-F | ACAGGACCATCGGAATCTTG |
|  | MX1-R | CCCTTCTTCAGGTGGAACAC |
| ***CxCL10*** | CxCL10-F | CTGCTTTGGGGTTTATCAGA |
|  | CxCL10-R | CCACTGAAAGAATTTGGGC |
| ***HMOX1*** | HMOX1-F | AGGGAAGCCCCCACTCAAC |
|  | HMOX1-R | ACTGTCGCCACCAGAAAGCT |
| ***NFE2L2*** | NFE2L2-F | CAGCGACGGAAAGAGTATGA |
|  | NFE2L2-R | TGGGCAACCTGGGAGTAG |
| ***BAX*** | BAX-F | TGGCAGCTGACATGTTTTCTGAC |
|  | BAX-R | TCACCCAACCACCCTGGTCTT |
| ***BCL2*** | BCL2-F | TCGCCCTGTGGATGACTGA |
|  | BCL2-R | CAGAGACAGCCAGGAGAAATCA |
| ***BCAT1*** | BCAT1-F | TGAGGCTTGGCTTTTGTGAA |
|  | BCAT1-R | GGCTCTGGTGTAACAAAGCC |
| ***HPRT*** | HPRT-F | GAACGTCTTGCTCGAGATGTG |
|  | HPRT-R | CCAGCAGGTCAGCAAAGAATT |
| ***HA*** | HA-F | CTCGTGCTATGGGGCATTCA |
|  | HA-R | TTCCAATCGTGGACTGGTGT |
| ***GSK3β*** | GSK3β-F | GGAACTCCAACAAGGGAGCA |
|  | GSK3β-R | TTCGGGGTCGGAAGACCTTA |
| ***MFN1*** | MFN1-F | GGTGAATGAGCGGCTTTCCAAG |
|  | MFN1-R | TCCTCCACCAAGAAATGCAGGC |
| ***OPA1*** | OPA1-F | GGCCAGCAAGATTAGCTACG |
|  | OPA1-R | ACAATGTCAGGCACAATCCA |
| ***FIS1*** | FIS1-F | GTCGACATGGAGGCCGTGCTGAAC |
|  | FIS1-R | CGGCCGTCAGGATTTGGACTTGGA |
| ***DRP1*** | DRP1-F | AGAAAATGGGGTGGAAGCAGA |
|  | DRP1-R | AAGTGCCTCTGATGTTGCCA |
| ***TFAM*** | TFAM-F | GGCAAGTTGTCCAAAGAAACC |
|  | TFAM-R | GCATCTGGGTTCTGAGCTTTA |
| ***MT-ND1*** | MT-ND1-F | CCACCTCTAGCCTAGCCGTTTA |
|  | MT-ND1-R | GGGTCATGATGGCAGGAGTAAT |
| ***GAPDH*** | GAPDH-F | GAAGGTGAAGGTCGGAGTC |
|  | GAPDH-R | GAAGATGGTGATGGGATTTC |
| ***DDX58*** | DDX58-F | AGTCTGACTGTCCTTTCTACTTGAAA |
|  | DDX58-R | ATCCCGTTGATCTCCAGGGAA |
| ***IFIH1*** | IFIH1-F | ATGGAAAAAAAAGCTGCAAAAGA |
|  | IFIH1-R | GTACTTCCTCAAATGTTCTGCACAA |
| ***SDHA*** | SDHA-F | AGCAAGCTCTATGGAGACCT |
|  | SDHA-R | TAATCGTACTCATCAATCCG |
| ***BCS1L*** | BCS1L-F | GACGGGTTACCCCAAACCAT |
|  | BCS1L-R | CACAGACAAAGCTCTCCCGT |
| ***COX5A*** | COX5A-F | AGGCTTAGGGGACTGGTTGT |
|  | COX5A-R | TAAGAGGGCTTGGCTACTGC |
| ***ATPAF2*** | ATPAF2-F | CCCAGCAGGATACCATCAAG |
|  | ATPAF2-R | TCCTAGCAGGCTGGATGAAC |
| ***GPX7*** | GPX7-F | AACTGGTGTCGCTGGAGAAG |
|  | GPX7-R | AAACTGGTTGCAGGGGAAG |
